# Separable neural mechanisms for the pleiotropic association of copy number variants with neuropsychiatric traits

**DOI:** 10.1038/s41398-020-0771-4

**Published:** 2020-03-13

**Authors:** Jonathan R. Reinwald, Alexander Sartorius, Wolfgang Weber-Fahr, Markus Sack, Robert Becker, Michael Didriksen, Tine B. Stensbøl, Adam J. Schwarz, Andreas Meyer-Lindenberg, Natalia Gass

**Affiliations:** 1 Department of Neuroimaging, Central Institute of Mental Health, Medical Faculty Mannheim, University of Heidelberg, Heidelberg, Germany; 2 Department of Psychiatry and Psychotherapy, Central Institute of Mental Health, Medical Faculty Mannheim, University of Heidelberg, Heidelberg, Germany; 3 H. Lundbeck A/S, Copenhagen, Denmark; 4 Takeda Pharmaceuticals, Cambridge, MA USA; 5 Department of Psychological and Brain Sciences, Indiana University, Bloomington, IN USA; 6 Department of Radiology and Imaging Sciences, Indiana University, Indianapolis, IN USA

**Keywords:** Molecular neuroscience, Medical genetics, Schizophrenia

## Abstract

22q11.2, 15q13.3, and 1q21.1 microdeletions attract considerable interest by conferring high risk for a range of neuropsychiatric disorders, including schizophrenia and autism. A fundamental open question is whether divergent or convergent neural mechanisms mediate this genetic pleiotropic association with the same behavioral phenotypes. We use a combination of rodent microdeletion models with high-field neuroimaging to perform a comparative whole-brain characterization of functional and structural mechanisms linked to high-risk states. Resting-state functional and structural magnetic resonance imaging data were acquired on mice carrying heterozygous microdeletions in 22q11.2 (*N* = 12), 15q13.3 (*N* = 11), and 1q21.1 (*N* = 11) loci. We performed network-based statistic, graph, and morphometric analyses. The three microdeletions did not share significant systems-level features. Instead, morphometric analyses revealed microcephaly in 1q21.1 and macrocephaly in 15q13.3 deletions, whereas cerebellar volume was specifically reduced in 22q11.2 deletion. In function, 22q11.2 deletion mice showed widespread cortical hypoconnectivity, accompanied by opposing hyperconnectivity in dopaminergic pathways, which was confirmed by graph analysis. 1q21.1 exhibited distinct changes in posterior midbrain morphology and function, especially in periaqueductal gray, whereas 15q13.3 demonstrated alterations in auditory/striatal system. The combination of cortical hypoconnectivity and dopaminergic hyperconnectivity and reduced cerebellum in 22q11.2 deletion mirrors key neurodevelopmental features of schizophrenia, whereas changes in midbrain and auditory/striatal morphology and topology in 1q21.1 and 15q13.3 rather indicate focal processes possibly linked to the emergence of abnormal salience perception and hallucinations. In addition to insights into pathophysiological processes in these microdeletions, our results establish the general point that microdeletions might increase risk for overlapping neuropsychiatric phenotypes through separable neural mechanisms.

## Introduction

There is an increasing consensus that the current categorical classification of neuropsychiatric disorders lacks biological validity^1^. In psychiatric genetics, this may contribute to repeated findings that both common and rare genetic variants are associated with a range of categorical diagnoses. A case in point are structural variations with relatively large segments of DNA deleted or duplicated, called copy number variants (CNVs), with deletions showing higher penetrance and more severe phenotypes than duplications^2^. Out of many existing deletions, specifically three microdeletions—22q11.2, 15q13.3, and 1q21.1—have been identified as high-penetrant and high-risk variants for neuropsychiatric phenotypes varying in severity and covering diverse syndromes, such as schizophrenia^2–4^, autism^5^, epilepsy, attention deficit hyperactivity disorder (ADHD), and intellectual disability^6^. These variants thus offer an excellent opportunity to determine the pathological pathways in these CNV constructs and to study the question whether pleiotropic association of different genetic variants with the same (set of) categorical phenotype is mediated by distinct or shared mechanisms, which would then be of interest as final pathways linked to genetic risk for that phenotype.

Modeling these microdeletions in animals is helpful in this context for the delineation of the essential systems-level features contributing to the neuropsychiatric traits. In humans, the severity of CNV phenotypes is strongly influenced by interactions with environmental adverse events or other genetic factors, including additional CNVs, mutations, and ethnicity^7^. Animal models offer higher inter-individual homogeneity, largely restricting influences from gene-environment interaction and other genetic risk factors, thus specifically focusing on the targeted genes. Further, most CNV clinical studies investigated disease-specific population and hence were biased by diagnosis-based pre-selection^8,9^. Finally, low incidence rates of microdeletions^2^ lead to relatively small sample sizes, limiting the ability to study their contributions to the etiology of resultant syndromes. For these reasons, mouse models of CNVs are extremely valuable tools for investigating the potential causes of neuropsychiatric traits associated with deletions.

One of the most studied CNVs is the microdeletion in the 22q11.2 region of chromosome 22. Consistently replicated data show its association with schizophrenia (odds ratio (OR) 9.3–492.8)^4^, autism spectrum disorder and ADHD^10^. From the six mouse models existing for this deletion^11–16^, Df(h22q11)/+ best represents the human deletion, being congenic and containing almost all functional genes of the human 1.5 Mb segment^16^. Mice carrying this deletion demonstrate behavioral deficits including reduced prepulse inhibition, increased acoustic startle response and impaired working memory^16^.

The other two microdeletions, 15q13.3 and 1q21.1, are far less investigated. The 15q13.3 deletion (seven genes) associates with schizophrenia (OR 3.7–66.5)^4^, epilepsy and autism^17^. Its mouse model expresses neurophysiological changes similar to humans, such as schizophrenia-like auditory processing deficits^18^, epileptic seizures^18^, and impairments in attention and memory^19^. The locus 1q21.1 (nine genes) associates with schizophrenia (OR 2.1–6.9)^4^, autism, ADHD, and seizures^20,21^. A recently created mouse model for this deletion is hypersensitive to dopamine-releasing drug amphetamine, resembling heightened psychostimulant sensitivity in schizophrenia^22^.

Most empirical approaches predominantly focused on behavioral, morphological, and local electrophysiological changes in these CNVs^16,18,19,22,23^, and, to the best of our knowledge, these data have not been systematically compared across variants. To accomplish this comparison, investigations of whole-brain functional connectivity (FC) and topology are useful, as dysfunctional connectivity linked to a disruption of information flow between distinct brain regions represents a transdiagnostic feature of psychiatric diseases, including schizophrenia, ADHD, and autism^24–26^, and can be readily compared across genetic groups or even species^27^. We profiled brain connectivity and morphology in 22q11.2, 15q13.3, and 1q21.1 deletion mouse lines to delineate systems-level brain changes, which could provide translational endophenotypes for understanding the pathophysiological mechanisms behind the liability to neuropsychiatric traits.

## Materials and methods

### Animals

All mouse lines were generated by TaconicArtemis (Köln, Germany) and shipped by Lundbeck (Valby, Denmark). Mouse deletion orthologous to human 22q11.2 is found at mouse 16qA3 chromosome, deletion orthologous to 15q13.3 - at 7qC chromosome, and deletion orthologous to 1q21.1 - at 3qF2.1 chromosome. The 10-week-old male mice carrying three different chromosomal deletions were investigated: deletion orthologous to 22q11.2 (Df(h22q11)/+ mice, *N* = 12, 22–26 g; wild type (WT) mice, *N* = 10, 22–27 g), 15q13.3 (Df(h15q13)/+ mice, *N* = 11, 23–28 g; WT mice, *N* = 12, 22–26 g), and 1q21.1 (Df(h1q21)/+ mice, *N* = 11, 21–25 g; WT mice, *N* = 11, 21–27 g). A group of WT littermates is a necessary condition, especially for the genetically modified mice, in order to have comparable genetic, epigenetic, and environmental backgrounds^28^. Thus, each mouse deletion group had its own littermate control group. Animals were bred by mating WT C57BL/6 N females with either hemizygous Df(h22q11)/+ males, Df(h15q13)/+ males, or Df(1q21)/+ males to avoid any placental or maternal care effects of the deletion.

Owing to the exploratory nature of this study, no formal power analysis for the sample size estimation was performed, but the number of animals per group (*n* = 11–12) were in the mid-range of sizes typically used in animal fMRI experiments.

All animals were group-housed (two WT and two hemizygous mice from the same litter per cage) under controlled conditions (19–23 °C, 40–60% humidity) on a 12 h dark/light cycle and underwent a two-week adaptation period between their arrival and the start of the MRI experiments.

All procedures were conducted according to the regulations covering animal experimentation within the European Union (European Communities Council Directive 86/609/EEC) and within the German Animal Welfare Act. Experiments were approved by the German animal welfare authorities (Regierungspräsidium Karlsruhe).

### MRI acquisition

All mice were of the same 10-week-old age by the time of MRI acquisition. Experiments were conducted at a 9.4 Tesla MRI scanner (94/20 Bruker Biospec, Ettlingen, Germany) with a two-element anatomically shaped cryogenic mouse surface coil (for details see Supplement). Anesthetic regime and recording of the physiological data were performed as previously^29^, including initiation of anesthesia with isoflurane, and using only medetomidine (0.8 mg/kg/h) for sedation (detailed description in Supplement). Breathing and cardiac rates were monitored and recorded. Group (deletion/WT) and time of day were randomized in the fMRI measurements. The investigator was blinded to the group allocation during the experiments.

To validate data quality and anesthesia effects on a broader, cross-laboratory level, we cooperated in a major international project comparing resting-state networks across 17 international imaging centers^30^. Our study demonstrated comparable and specific default-mode network connectivity (Supplementary Fig. 8 in ref. ^30^).

The rs-fMRI time series were acquired using an echo-planar imaging (EPI) sequence with the following parameters: repetition time (TR)/echo time (TE) 1300/18 ms, flip angle 50°, 21 slices, 96 × 64 matrix, field of view (17.28 × 11.52) mm^2^, slice thickness 0.4 mm, 400 acquisitions. Four initial volumes were included as dummy scans in the pre-scan protocol, but were not considered in the analyses, to avoid magnetization influences before the scanner achieves steady state. Magnetic field (B0) inhomogeneity was measured with Bruker FieldMap sequence (TR = 20 ms, short TE = 1.7 ms, long TE = 5.7 ms) acquired before each EPI. Measured field values were then used in preprocessing to calculate the geometric distortion and signal loss for the compensation of these artefacts.

Structural data were acquired using rapid acquisition with refocused echoes (RARE) sequence (RARE factor 16, TR/TE 1200/50 ms, flip angle 180°, 225 × 192 × 96 matrix, field of view (17.5 × 15 × 15) mm^3^, acquisition time 23 min).

### Deformation- and voxel-based morphometry (DBM and VBM)

DBM was performed using SPM12 (http://www.fil.ion.ucl.ac.uk/spm/software/spm12) and in-house scripts developed in Matlab R2017a (MathWorks Inc., USA) (for details, see Supplement). Preprocessing included coregistration, brain extraction, segmentation based on in-house high-resolution tissue probability maps^31^ and application of Diffeomorphic Anatomical Registration using Exponentiated Lie algebra (DARTEL) tool^32^ to obtain animal-specific Jacobian determinant maps representing transformation of individual animal maps to the average-shaped template. Brain extraction was performed using an in-house tool with an adapted algorithm based on a 3D pulse-coupled neural network^33^, incorporating prior data optimization including contrast and edge enhancement and intensity normalization (Figure S1). Afterwards, we additionally checked every brain for the quality of extraction (for details, see Supplement). To assess differences in total brain volume, mean values of Jacobian determinants were calculated and compared between each deletion and its respective WT group using two-sample *t* tests. Region of interest (ROI) volumetry analysis was used to assess regional differences within and between the CNVs. The volumes of each region were calculated as a mean value per pre-defined ROI, based on the individual Jacobian determinant maps. To account for differences in total brain volume, regional volumes were transformed into percentages of the individual total brain volume. These relative values were compared between each deletion group and its respective control using two-sample *t* tests. Further, between-CNV similarities and differences were assed using analysis of variance (ANOVA).

### Region of interest

**Fig. 1:**
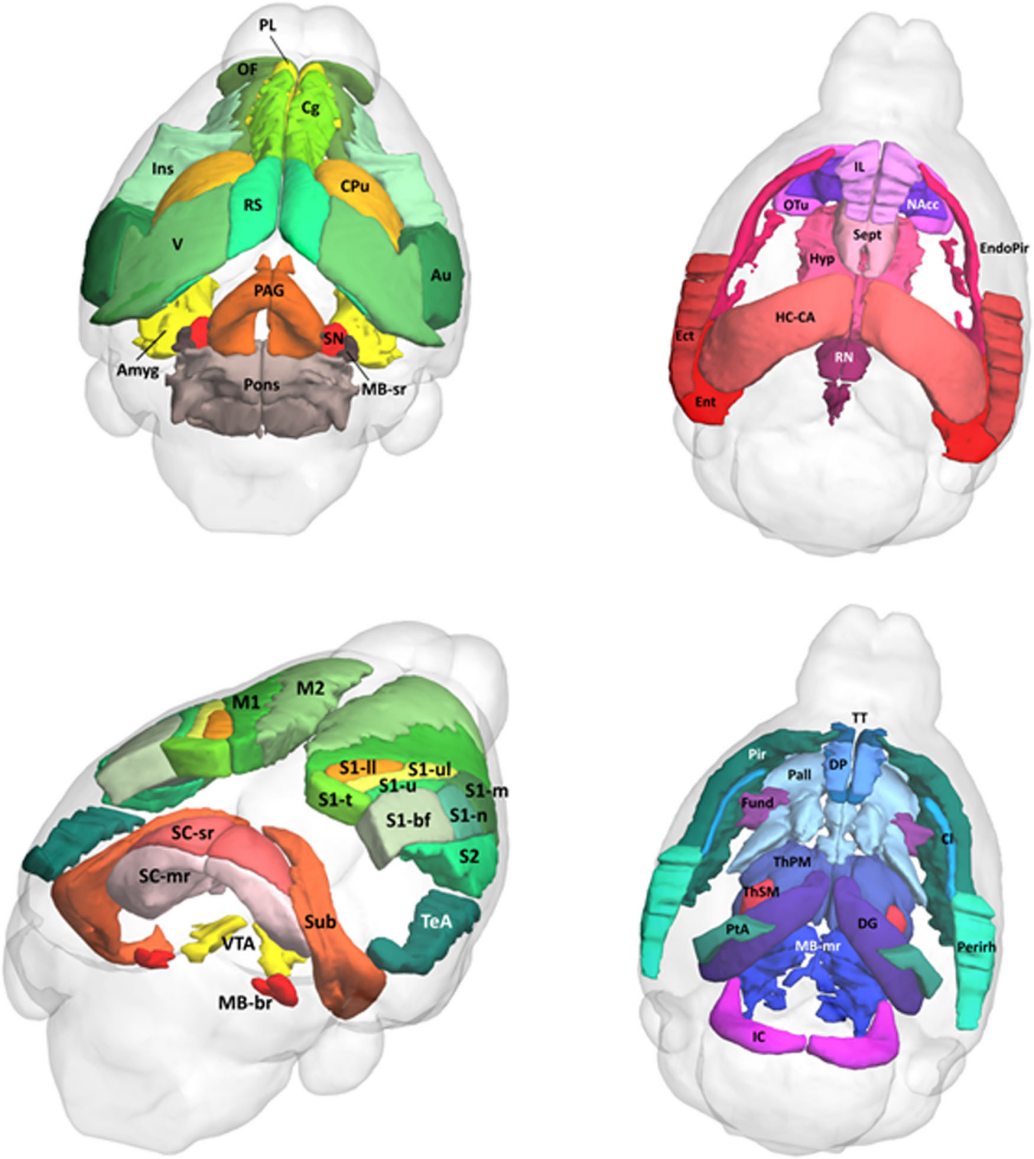
Representation of 104 regions of interest from Allen Mouse Brain Atlas^34^ used in the network-based statistics and graph theoretical analyses. Regions of interest were normalized to the mouse atlas template in the Paxinos space^35^. *Amyg* amygdala, *Au* auditory cortex, *Cg* anterior cingulate area, *Cl* claustrum, *CPu* caudate-putamen, *DG* dentate gyrus, *DP* dorsopeduncular cortex, *Ect* ectorhinal cortex, *EndoPir* endopiriform nucleus, *Ent* entorhinal cortex, *Fund* fundus of striatum, *HC-CA* hippocampus Ammon’s horn, *Hyp* hypothalamus, *IC* inferior colliculus, *IL* infralimbic cortex, *Ins* insular cortex, *M1* primary motor cortex, *M2* secondary motor cortex, *MB-br* midbrain behavior-related, *MB-mr* midbrain motor-related, *MB-sr* midbrain sensory-related, *NAcc* nucleus accumbens, *OF* orbitofrontal cortex, *OTu* olfactory tubercle, *PAG* periaqueductal gray, *Pall* pallidum, *Perirh* perirhinal cortex, *Pir* piriform cortex, *PL* prelimbic cortex, *PtA* parietal association cortex, *RN* raphe nuclei, *RS* retrosplenial cortex, *S1-bf* primary somatosensory cortex barrel field, *S1-ll* S1 lower limb, *S1-m* S1 mouth, *S1-n* S1 nose, *S1-t* S1 trunk, *S1-u* S1 unassigned, *S1-ul* S1 upper limb, *S2* secondary somatosensory cortex, *SC-mr* superior colliculus motor-related, *SC-sr* superior colliculus sensory-related, *Sept* lateral septal complex, *SN* substantia nigra, *Sub* subiculum, *TeA* temporal association cortex, *ThPM* thalamus polymodal association cortex related, *ThSM* thalamus sensory-motor cortex related, *TT* tenia tecta, *V* visual cortex, *VTA* ventral tegmental area.

For subsequent structural and functional analyses, 104 unilateral regions were selected from the Allen Mouse Brain Atlas (Fig. 1)^34^ using an in-house interactive tool (written in MATLAB), which includes the automatic transformation and normalization of the selected ROIs from the Allen atlas space to the standard mouse atlas template in the Paxinos space^35^. For a detailed illustration of ROI selection and normalization, see Figure S2 in the Supplement.

### Preprocessing of rs-fMRI data

Preprocessing was done as previously^36^ and included the following steps: correction for magnetic field inhomogeneities (SPM12), filtering of respiratory and cardiac signals (Aztec software^37^), slice timing correction (SPM12), spatial normalization, and coregistration to a mouse brain template in the Paxinos stereotactic coordinate system^35^, regression of movement parameters and cerebrospinal fluid signal (FSL, version 4.1. http://www.fmrib.ox.ac.uk/fsl). Aztec is a well-established and highly valid cardiorespiratory correction software for functional MRI data regressing confounds and fluctuations owing to cardiac pulsation and heart rate based on RETROICOR^37^. Calculating maps of explained variance by the physiological parameters, it provides a region-specific noise correction, illustrated in Figure S3. For a detailed statistical analysis of physiological parameters and a more profound description of physiological noise correction, see Supplement. The normalization included three steps: (1) linear coregistration (six degree-of-freedom rigid-body transformation) to individual 3D structural datasets (without reslicing), (2) non-linear spatial normalization (estimate & write) of 3D datasets to the atlas template, (3) normalization (write) of EPI datasets to the atlas using transformation matrix from step (2).

An additional method identifying motion-affected frames based on a decomposition of DVARS, the spatial root mean square of the data after temporal differencing^38^, was integrated to further remove motion artefacts (Figure S4). This method enables removal of motion-affected scans based on *p* values instead of an arbitrary threshold with subsequent scrubbing using linear interpolation^39^. Finally, bandpass filtering was performed (0.01-0.1 Hz, Analysis of Functional NeuroImages (AFNI) software^40^).

For subsequent functional analyses, one animal (15q13.3, WT) was excluded owing to high motion affecting > 10% of all time-frames. Further, four animals classified as outliers by comparing the mean FC (the standard deviation more than double compared with the mean) were excluded (*N* = 1 WT of Df(h22q11)/+; *N* = 1 Df(h15q13)/+; *N* = 2 Df(1q21)/+ and its WT) (Figure S5).

### Network-based statistics (NBS)

Mean BOLD time courses were extracted from 104 unilateral ROIs. Pearson’s correlation coefficients were computed pairwise between all regional time courses for each animal, resulting in individual correlation matrices, with brain regions forming nodes and correlation coefficients forming edges. NBS, a non-parametric cluster-based method aiming to identify any potential connected structure formed by a set of supra-threshold links^41^, was performed to assess FC differences between the CNV groups and controls. The statistical model was specified in terms of a general linear model with *F* tests performed for every edge. A primary threshold (*F*_21_ = 8.18 to *F*_20_ = 8.29, *p*_pt_ < 0.01) was used to discard sub-threshold edges, and the contiguous surviving edges were defined as cluster. The extent of the resulting cluster was compared to the maximum extents of clusters resulting from 5.000 random permutations (*p*_NBS_ < 0.05 for individual permutation test).

### Graph theoretical analyses

Although network-based statistics applies a special cluster statistics^41^ to quantify widespread group differences of a connectivity graph, graph theoretical analysis characterizes brain networks with a number of neurobiologically meaningful and easily computable measures^42^. These measures variously detect aspects of functional integration and segregation, quantify importance of individual brain regions, or characterize patterns of local anatomical circuitry (see Supplement for details).

#### Network construction and density

Network construction and graph metric calculation was done as previously^36^. The Pearson’s correlation matrices were normalized by their maximum weights and used to compute FC graphs. Topological characteristics of FC networks were calculated using Brain Connectivity Toolbox (version 2016-01-16)^42^. We focused on a 16–50% range (1% step) of network densities for calculating metrics using binarized networks. We ensured that networks maintained connectedness, meaning the ability of every node to reach every other node. The range of density thresholds was comparable to those of several studies assessing network alterations in humans^43,44^ and animals^27,36^. Areas under the curve calculated as the average of this range were used to focus the analysis toward identifying systematic effects that are not strongly dependent on a specific threshold.

#### Graph metrics for assessing regional and global architecture

To explore changes in global topology, we calculated small worldness, global clustering coefficient, characteristic path length and modularity (for details, see Supplement). Modular partitions of the individual networks were detected using the Newman algorithm^45^.

Degree and local clustering coefficient were assessed to explore regional alterations in network structure. Normalization was done using comparable random graphs preserving number of nodes, degree distribution, and connectedness as null models^46^. Group comparison was performed using two-sample *t* test (two-sided, *p* < 0.05) with age as a covariate.

### Amplitude of low frequency fluctuations (ALFF)

ALFF^47^ quantifies the amplitude of low frequency oscillations, a fundamental feature of the resting brain^48^. After preprocessing including bandpass filtering, time series were transformed to a frequency domain using Fast Fourier transform and the power spectrum was obtained^47^. ALFF was calculated as the averaged square root of the power spectrum at each voxel, was *z-*transformed, and then mean values for each ROI were computed. Group comparison was performed using two-sample *t* tests (two-sided, *p* < 0.05).

### Comparison between deletions

Differences in whole-brain connectivity between CNV deletions were assessed using NBS with ANOVA focusing on interaction effects between group (WT or deletion) and deletion (type of deletion) (for details, see Supplement). Similarly, interaction terms of ANOVA were assessed to compare graph metrics and ALFF between the CNV groups, with post hoc analyses to compute the direction of the differences. To complement local topological findings with regional FC alterations, which might not be caught by a whole-brain approach like NBS, we performed seed-based analyses for selected brain regions (for details, see Supplement).

## Results

### Structural analyses

**Fig. 2:**
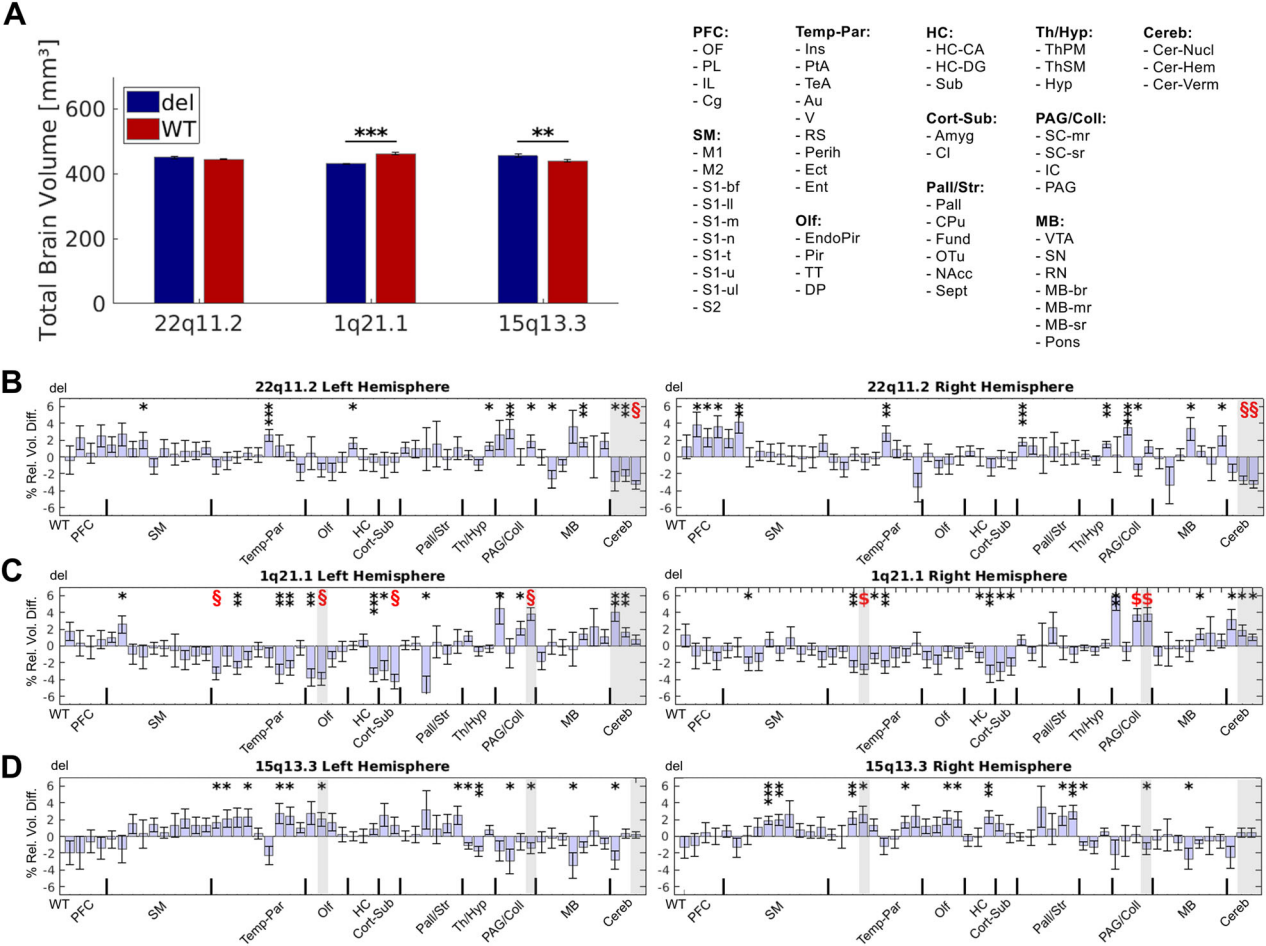
Total and regional brain volume differences between Df(h22q11)/+, Df(h1q21)/+, and Df(h15q13)/+ mice and their respective controls. Bars (mean) with error bars (standard error of the mean) illustrate total **a** and regional **b**–**d** alterations in the brain volume. **a** Deformation-based morphometry revealed a significantly smaller total brain volume in Df(h1q21)/+ mice compared with their controls, whereas Df(h15q13)/+ mice exhibited a significantly larger total brain volume. Regional volume changes between the three CNVs and their littermate controls illustrated in **b**–**d** demonstrate the percentage difference of the relative regional brain volumes (% of respective total brain volume) of the CNV compared with its WT control allowing to interpret the results as focal alterations independent from the whole-brain changes caused by the CNVs. Shadowed areas in **b**–**d** illustrate between-CNV differences (in relation to the respective WT) revealed by the ANOVA interaction effects (*p* < 0.05, Bonferroni-corrected for 104 brain regions and number of tests). % rel. vol. diff., percent relative regional volume difference; WT, wild type; del, deletion; *, significant at *p* < 0.05; **, significant at *p* < 0.01; ***, significant at *p* < 0.001; §, significant at *p* < 0.05 Bonferroni-corrected for 104 brain regions.

Pairwise *t* tests of the total brain volume revealed a highly significant microcephaly (*p* < 0.001) in Df(h1q21)/+ mice, whereas Df(h15q13)/+ mice demonstrated a macrocephaly (*p* < 0.01, Fig. 2a). Additional ROI volumetry analysis allowed a comparison of regional relative brain volumes corrected for the whole-brain differences between and within the CNV groups. Importantly, Df(h22q11)/+ mice demonstrated significantly smaller cerebellar regions compared with its controls (Fig. 2b, *p* < 0.05, Bonferroni-corrected). Macrocephalic regions in Df(h1q21)/+ mice were predominantly located in the temporo-parietal, hippocampal, olfactory, and subcortical regions with several of them surviving multiple comparison correction (Fig. 2c, *p* < 0.05, Bonferroni-corrected) and affecting among others insular, auditory, and piriform cortex. Of note, posterior brain regions including periaqueductal gray (PAG), inferior colliculus, and cerebellum demonstrated opposing findings of enlarged volumes (Fig. 2c, *p* < 0.05, Bonferroni-corrected). The Df(h15q13)/+ mice had larger volumes of the sensory-motor, temporo-parietal and subcortical brain areas, however none of them surviving multiple comparison correction (Fig. 2d).

Between-group investigations using ANOVA yielded highly significant differences (Fig. 2b–d, shadowed areas) between cerebellar regions of Df(h22q11)/+ in contrast to Df(h15q13)/+ and Df(h1q21)/+ mice (*p* < 0.05, Bonferroni-corrected). Further, PAG and piriform cortex showed contrasting alterations in size between Df(h1q21)/+ and Df(h15q13)/+ mice. Our analyses did not detect any similarities between the CNV deletions in relation to their respective controls (*p* > 0.05, Bonferroni-corrected). The effect sizes, correlations to body weight and ventricular volumes differences are presented in Supplement (Figures S6, S7, S8).

### Amplitude of low frequency fluctuations

Importantly, only very few brain regions showed significant differences between the deletion group and the WT control (Figure S9, *p* < 0.05, uncorrected), with none of them surviving multiple comparison correction (*p* > 0.05, Bonferroni-corrected). While we could find only marginal differences in 1q21.1 and 15q13.3 deletions, regional differences were slightly more pronounced in 22q11.2 deletion, predominantly located in cortical, somato-motor and subcortical brain areas (*p* < 0.05, uncorrected).

### Network-based statistics

**Fig. 3:**
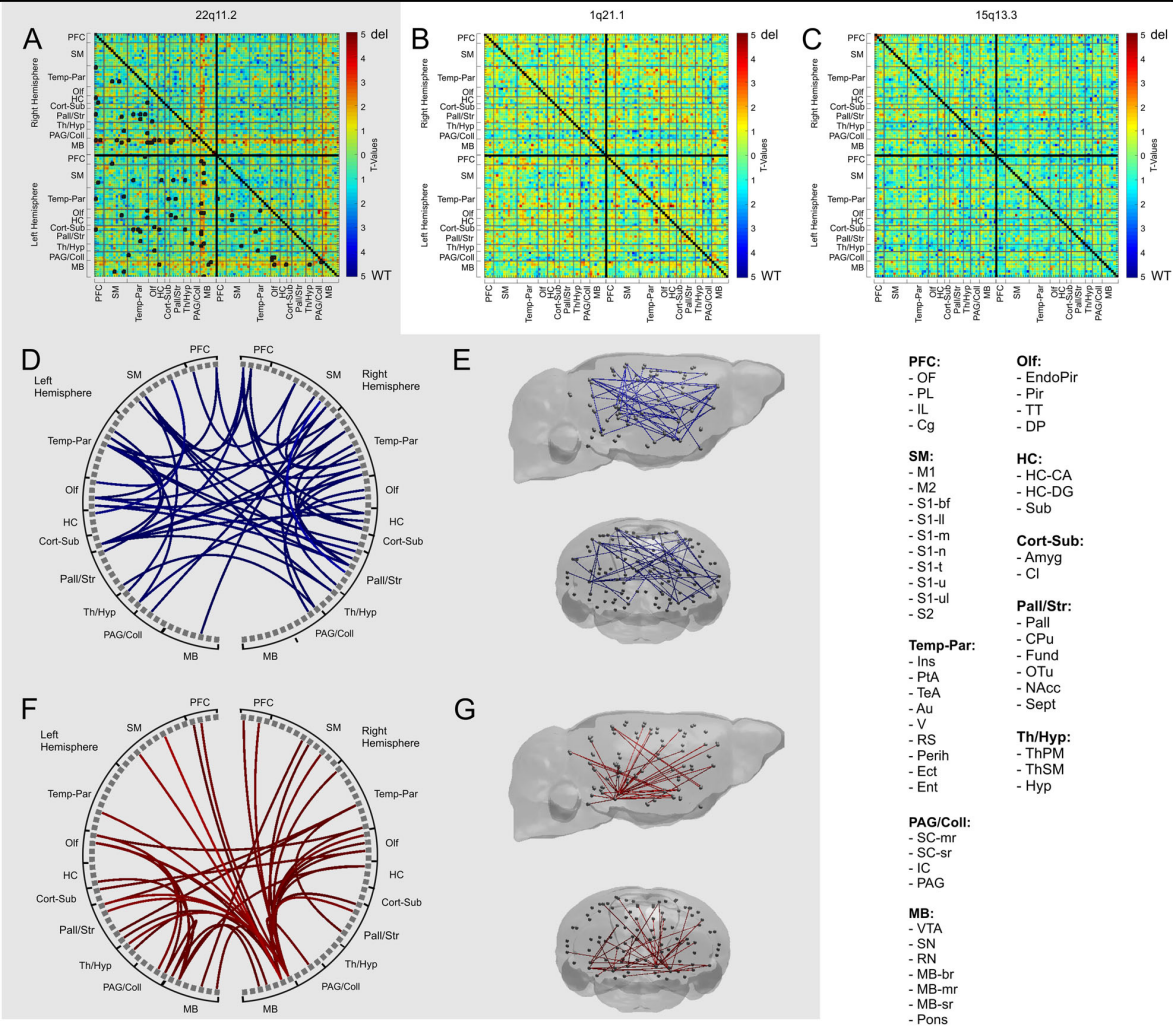
Differences in functional connectivity (FC) between Df(h22q11)/+, Df(h1q21)/+, and Df(h15q13)/+ mice and their respective controls, detected by the network-based statistics (NBS) analyses. The gray-shaded area represents results for the 22q11.2 deletion. **a** NBS analysis revealed a widespread cluster of FC differences in the Df(h22q11)/+ mice compared with its control (*p*_pt_ < 0.01, *p*_NBS_ < 0.05). Black boxes in the matrix represent the connections within the significant cluster at *p* < 0.05. **b, c** No significant group differences were detected between Df(h1q21)/+ or Df(h15q13)/+ mice and their respective controls in NBS analysis (*p*_NBS_ ≥ 0.05). **d, e** The pattern of decreased FC in 22q11.2 deletion covered predominantly cortical brain regions. **f, g** The pattern of increased FC in 22q11.2 deletion was mostly restricted to mesocortical and mesolimbic pathways from the midbrain, with ventral tegmental area (VTA) and substantia nigra (SN) as main hubs. WT, wild type; del, deletion; PFC, prefrontal cortex; SM, sensory-motor areas; Temp-Par, temporo-parietal areas; PAG/Coll, periaqueductal gray and colliculi; Olf, olfactory areas; HC, hippocampal areas; Cort-Sub, cortical subplate; Pall/Str, pallidum and striatum; Th/Hyp, thalamus and hypothalamus. For the abbreviations of individual brain regions see legend for Fig. 1.

NBS analyses detected a widespread cluster in the Df(h22q11)/+ group, consisting of two elements with opposing FC alterations (*p*_pt_ < 0.01, *p*_NBS_ < 0.05, Fig. 3a). Decreased FC predominantly occurred in the cortical (prefrontal cortex and temporo-parietal cortex), hippocampal and striatal areas (Fig. 3d), affecting cortico–cortical, cortico-hippocampal, and cortico–striatal connections (Fig. 3e). In contrast, the pattern of increased FC was restricted to the mesocortical and mesolimbic pathways, with the ventral tegmental area (VTA) and substantia nigra (SN) forming the main hubs (Fig. 3f, g).

No significant group differences appeared in Df(h1q21)/+ and Df(h15q13)/+ mice, when compared with their controls (*p*_NBS_ ≥ 0.05, Fig. 3b, c). Also, no common connection was altered in all three CNVs relative to their respective controls (Figure S10).

### Graph analysis

None of the global metrics showed significant deletion vs. WT differences (*p* ≥ 0.05, Figure S11). However, regional analyses revealed more subtle and focal changes in network structure.

**Fig. 4:**
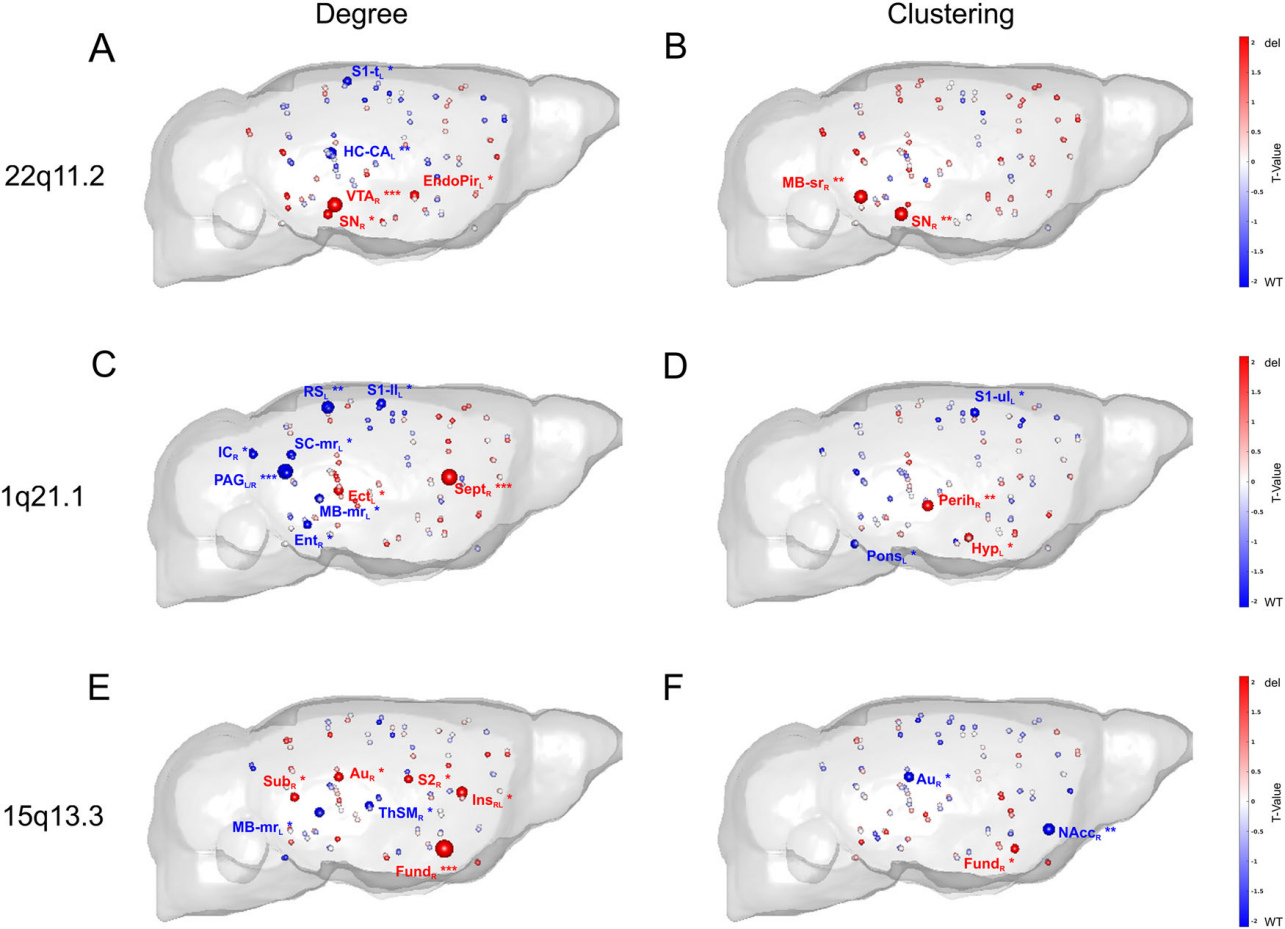
Differences in local graph metrics between 22q11.2, 1q21.1, and 15q13.3 deletion mice and their respective controls. Area under the curve T-values for degree **a**–**e** and local clustering coefficient **b**–**f** calculated for network densities from 16% to 50% are illustrated in the brains for 22q11.2, 1q21.1, and 15q13.3 deletion mice compared with their respective WT controls. Sphere colors and sizes are coded by *T* values. *, significant at *p* < 0.05; **, significant at *p* < 0.01; ***, significant at *p* < 0.001. WT, wild type; del, deletion. For the abbreviations of individual brain regions see legend for Fig. 1.

In Df(h22q11)/+ mice, local graph metrics supported our NBS observations of increased FC of midbrain regions by demonstrating higher degree (Fig. 4a) and local clustering (Fig. 4b) for VTA (*p*_uncorr_ < 0.001), SN (*p*_uncorr_ < 0.05 for degree; *p*_uncorr_ < 0.01 for clustering), sensory-related midbrain nuclei (*p*_uncorr_ < 0.01), and endopiriform cortex (*p*_uncorr_ < 0.05). In contrast, primary somatosensory cortex (*p*_uncorr_ < 0.05) and hippocampus (*p*_uncorr_ < 0.01) exhibited decreased degree. Local clustering tended to be higher in Df(h22q11)/+ mice for almost all brain regions, but this tendency was not reflected in a significant global clustering difference.

In contrast to Df(h22q11)/+ mice, Df(h1q21)/+ group exhibited a significant decrease of degree in multiple posterior midbrain regions, including PAG (*p*_uncorr_ < 0.001), inferior colliculus (*p*_uncorr_ < 0.05), superior colliculus (*p*_uncorr_ < 0.05) and midbrain motor-related (*p*_uncorr_ < 0.05) (Fig. 4c). Fewer regions, predominantly located in the frontal or parietal part (septal nuclei and ectorhinal cortex, *p*_uncorr_ < 0.05), showed higher degree.

In Df(h15q13)/+ mice, the overall effects on local metrics were weaker. Although degree was higher for cortical, striatal and hippocampal regions (fundus of the striatum, *p*_uncorr_ < 0.001; insular, *p*_uncorr_ < 0.05; auditory cortex, *p*_uncorr_ < 0.05; secondary somatosensory cortex, *p*_uncorr_ < 0.05; subiculum, *p*_uncorr_ < 0.05), sensory-motor thalamus and midbrain motor-related exhibited decreased degree (*p*_uncorr_ < 0.05). Equally, no clear tendency was found for clustering, with only few significant alterations in the nucleus accumbens, striatum and auditory cortex.

No joint effect of all CNV deletions vs. WT for global measures and no nominally significant effect surviving multiple comparison correction for local measures were detected.

### Comparison between deletions

Comparisons on a global level (NBS and global graph metrics) revealed no significant differences between the CNV deletions (ANOVA, *F* test for group x CNV interaction, *p* ≥ 0.05).

On a local level, ANOVA with post hoc tests corroborated our findings of midbrain structures being embedded with opposing valence into network topology in the 22q11.2 and 1q21.1 CNV groups. The higher degree in Df(h22q11)/+ mice and lower degree in Df(h1q21)/+ group were significant for bilateral inferior colliculus and bilateral PAG (Figure S12). Comparable alterations were found for clustering coefficient of the PAG and sensory-related midbrain nuclei (Figure S13).

Seed-based analyses, performed to complement local topological findings, corroborated our graph metrical results by demonstrating higher FC of inferior colliculus, PAG, VTA, and SN in Df(h22q11)/+ compared with Df(h1q21)/+ and Df(h15q13)/+ mice (Figure S14).

## Discussion

Our structural and functional MRI investigation of Df(h22q11)/+, Df(h15q13)/+, and Df(h1q21)/+ mouse lines identified significant, but clearly distinct systems-level effects. A pronounced pattern of cortical hypoconnectivity and midbrain hyperconnectivity in 22q11.2 deletion suggests these macroscopic circuit disruptions as a mechanism behind the particularly high genetic liability to different neuropsychiatric traits in this deletion. We confirmed these results in graph analysis by revealing differential embedding of midbrain dopaminergic regions into brain topology. In structure, a prominent reduction of cerebellar volume implies a distinct role of the cerebellum in the pathomechanism of 22q11.2 deletion. As both findings represent key features of schizophrenia, our study provides a straightforward explanation for the high incidence of schizophrenia in 22q11.2 microdeletion carriers. Further, by demonstrating differences in regional brain morphology and local graph metrics between Df(h22q11)/+, Df(h15q13)/+, and Df(h1q21)/+ groups, our study supports the idea that each CNV contributes via a specific and divergent mechanism to the risk for neuropsychiatric disabilities. Even at an exploratory *p* < 0.05 uncorrected threshold, no common connections were altered in all three CNVs relative to their controls.

### Brain structural abnormalities

We found a decrease of total brain volume in 1q21.1 and an increase in 15q13.3 deletion. Consistently, several works demonstrated smaller brain sizes in children and adults with 1q21.1 deletion syndrome^20,21^, being put in the context of the well-established findings of lower gray matter volume and microcephaly in patients with schizophrenia^20^. Our study is the first report of microcephaly in a mouse model of this deletion.

Our opposing finding of macrocephaly in 15q13.3 deletion confirms previous animal report^49^. Interestingly, 15q13.3 deletion enhances risk for autism^5,50^ associated with macrocephaly^51^. This endophenotype might partially result from reduced synaptic pruning described in children and adolescents with autism^52^. However, such linkage must be considered with caution, as associations between alterations in total brain volume and complex neuropsychiatric diseases probably oversimplify the relationship between brain structure and disease, only reflecting a small part of the multiple risk factors interacting during the psychopathology development. This aspect might also explain why we discovered no brain volume differences in 22q11.2 deletion despite its prominent neuropsychiatric phenotypes^4^.

ROI volumetry analysis provides a more detailed regional investigation and depicts brain size focal changes potentially reflecting more subtle effects caused by CNV deletions. In Df(h22q11)/+ mice, we found a highly significant cerebellar volume reduction being in perfect agreement with recent findings of a multi-center mega-analysis of 983 patients with schizophrenia^53^. As their findings were consistent across the included ages, they suggested a neurodevelopmental etiology to be more probable than a neurodegenerative^53^, to which CNV deletions might contribute. Importantly, the results of reduced cerebellar volume were highly specific for 22q11.2 deletion (*p* < 0.05, Bonferroni-corrected), suggesting it as a distinct morphological link between this CNV and its extraordinarily high risk for schizophrenia.

In contrast, reductions of brain volume in 1q21.1 deletion were focused on temporo-parietal and subcortical areas, accompanied by increased sizes of inferior colliculus, PAG, and cerebellar volume. As these alterations differed significantly from the other CNVs (*p* < 0.05, Bonferroni-corrected), they might result from a specific morphological mechanism. This is of certain interest, as PAG also demonstrated between-group graph analytical differences for this CNV, suggesting that it plays a key role in the 1q21.1 deletion risk for psychiatric disorders.

Focally enlarged regional volumes in 15q13.3 deletion were widespread throughout various neocortical areas, but were weaker in general. Importantly, the lack of strong similarities for regional volume alterations among all CNVs supports the idea of separable neural mechanisms rather than convergent pathological pathways.

### Brain functional abnormalities

#### 22q11.2

Initially focusing on whole-brain FC alterations, we detected robust widespread changes in 22q11.2 deletion and no differences in the other two groups. The cluster of altered FC consisted of two components with opposing changes: (1) decreased cortico–cortical, cortico–hippocampal, and cortico–striatal FC, and (2) increased FC within the mesocortical and mesolimbic pathways, particularly from VTA and SN. Importantly, these whole-brain connectivity alterations are consistent with previous studies of 22q11 deletion^54–56^ and schizophrenia^57^, and hence provide a potential mechanistic background for 22q11.2 extraordinarily high ORs for schizophrenia^4^.

Similar to our finding, Sigurdsson et al.^54^ demonstrated drastically reduced hippocampal-prefrontal neural synchrony in Df(h22q11)/+ mice. Aberrant connectivity within this circuit is common for many neuropsychiatric disorders possibly underlying cognitive impairment and emotional dysregulation^58^. Hypoconnectivity within sensory, fronto-parietal, and visual circuits is consistent with recent results from animal^59,60^ and clinical studies of 22q11.2 deletion, revealing reduced top–down fronto-temporal control^56,61^ and decreased intrinsic connectivity within auditory and visual regions^61^ postulated to underlie psychotic symptoms^59,60^.

Converging evidence points towards dopaminergic alterations in 22q11.2 deletion^16,62,63^ that are in accordance with both our NBS findings of hyperconnectivity for VTA and SN and their graph analytical results of higher degree and clustering. Specifically, patients with 22q11.2 deletion syndrome exhibit a hyperdopaminergic state with severe SN alterations^62^ hypothesized to precede the onset of dopaminergic degeneration at later stage, ultimately resulting in higher risk for Parkinson’s disease in elderly deletion carriers. The 22q11.2 deletion includes *COMT* gene, encoding an enzyme catalyzing inactivation of catecholamines, thereby providing a mechanistic explanation for the hyperdopaminergic state. Indeed, Df(h22q11)/+ mice exhibit increased dopamine metabolites’ levels in the prefrontal cortex and striatum^16^ and abnormal dopaminergic modulation^63^. Hemizygous *PRODH* gene deletion, also located on 22q11.2, provides further background for the hyperdopaminergic hypothesis, as *Prodh*-deficient mice have increased glutamate release which enhances dopaminergic signaling^64^. Altogether, our results point to differences in the embedding of VTA and SN into whole-brain topology. As these regions also demonstrated differences between deletions in the complementary seed-based analysis, we propose altered FC of VTA and SN as a mechanism conferring risk of neuropsychiatric traits specifically in 22q11.2 deletion.

#### 1q21.1

In Df(h1q21)/+ mice, local graph metrics demonstrated a remarkable pattern of decreased degree and clustering in posterior midbrain regions including PAG and colliculi. Being primarily acoustic and visual centers, the colliculi integrate sensory information of aversive nature and participate in defensive behavior, along with the PAG, another component of the encephalic aversion system^65,66^. Therefore, these graph analytical changes should be seen in the context of anxiety and altered sensory processing, which are transdiagnostic features of various neuropsychiatric syndromes including autism^67^ and schizophrenia^68^. Also, the pattern of altered midbrain connectivity was significantly different between Df(h1q21)/+ and Df(h22q11)/+ mice for both graph analytical and seed-based investigations, thus representing a specific pathogenic pathway of 1q21.1 deletion.

#### 15q13.3

In comparison with other CNVs, Df(h15q13)/+ mice exhibited less clear patterns of altered local graph metrics. Changes mainly occurred in parts of the ventral striatum and auditory cortex. Differential network embedding of ventral striatal areas might underlie deficits in decision making and reward processing common across neuropsychiatric diseases^69^. Alterations in the auditory cortex might explain decreased auditory evoked potentials^18,23,49^ and reduced acoustic startle response^70^ reported in 15q13.3 deletion. These changes point towards deficiencies in auditory perception hypothesized to underlie acoustic hallucinations in schizophrenia^71^ and impaired auditory processing in autism^72^.

### Limitations

As the study was conducted on sedated animals, functional data obtained from awake mice would be useful to confirm the presented findings. However, as medetomidine induces only a mild sedation compared with general anesthesia, and evokes stable and robust BOLD response and respiratory and cardiac activity^73,74^, we do not expect the results to be drastically different. Although the number of animals per group (*N* = 10–12) was in the mid-range typically used in animal fMRI experiments^75,76^, it might not have been sufficient for detecting subtle alterations in brain function. Finally, a recent investigation in 15q13.3 deletion demonstrated an importance of exposure to stress to induce a severe phenotype^77^. This interaction between the microdeletion and adverse environmental event might shape a more pronounced endophenotype. Although we did not explicitly expose our animals to distinct severe stressors, shipment and scanning procedures could be powerful stress factors.

## Conclusion

With the aim of determining pathological pathways in the CNV mouse constructs, we identified two specific features in Df(h22q11)/+ mice, suggesting them as mechanisms behind the particularly high genetic liability to neuropsychiatric traits in this deletion. Functionally, a pronounced pattern of cortical hypoconnectivity and midbrain hyperconnectivity in Df(h22q11)/+ mice stands out, whereas structurally a CNV-specific decrease in the cerebellar volume implies a key role of the cerebellum in the pathophysiology. Changes in the midbrain morphology and topology in Df(h1q21)/+ and auditory system in Df(h15q13)/+ mice provide insights for distinct pathophysiological mechanisms in these CNVs. Interestingly, 1q21.1 and 15q13.3 deletions, representing weaker risk factors for neuropsychiatric traits, also had more circumscribed functional effects. Thus, although brain structural and functional analyses showed significant alterations plausibly linked to the observed behavioral abnormalities and neuropsychiatric categories, they did so through separable mechanisms, indicating that different gene sets can impact different systems-level pathophysiological processes to increase risk for the same (set of) categorical phenotypes^2^.

## Supplementary information

Supplementary
