# Supplementary material for: Separable neural mechanisms for the pleiotropic association of copy number variants with neuropsychiatric traits
Source: Transl Psychiatry. 2020 Mar 13;10:93. doi: 10.1038/s41398-020-0771-4 (PMC7069945; doi:10.1038/s41398-020-0771-4)
Supplement: Supplementary file 1 — Supplementary [file 41398_2020_771_MOESM1_ESM.docx]

**Supplemental Information**

**Methods and Materials**

**MRI acquisition**

Anesthesia was induced at 4% isoflurane in a mixture of 70% N_2_ and 30% O_2_. When positioning mouse in the scanner (head first, prone), we reduced isoflurane concentration to ~1.5%. A bolus of medetomidine (0.4 mg/kg, 0.2 ml) was injected subcutaneously. Then isoflurane was gradually discontinued (starting 2 min after bolus and then reduced by 0.2% every min). At 0.3% isoflurane, a continuous infusion of medetomidine at 0.8 mg/kg/h was initiated, and thereafter isoflurane was switched off. Breathing and cardiac rates were monitored using a respiration pad placed beneath the chest (Small Animal Instruments Inc., NY, USA) and a pulse oximeter attached to the tail. Signals were recorded (10-ms resolution) during EPI acquisition using a signal breakout module (Small Animal Instruments Inc., NY, USA) and a 4-channel recorder (Velleman N.V., Gavere, Belgium) together with the scanner trigger pulses for each measured brain volume.

**Brain extraction**

Skull-stripping was done using an algorithm based on a 3D pulse-coupled neural network (3D-PCNN) (1) which is a well-established and effective tool (2) that has been proven to be valid for brain extraction tasks in rodents. Briefly, PCNNs are neural network models developed to model the observed synchronization of neural assemblies and are used for high-performance image processing without training data (3). Our in-house tool integrated prior data optimization including contrast and edge enhancement and intensity normalization. Additionally, it provides a stepwise visual control of brain extraction to refine the automatically calculated optimum.

**Region of interest (ROI) definition**

**Figure S2** illustrates the workflow including ROI selection and transformation to the Dorr atlas template in the Paxinos space in comparison to the normalization of our structural images.

**Deformation- and voxel-based morphometry (DBM and VBM)**

High resolution structural images were brain-extracted and co-registered to a template (4) in Paxinos standard space. In-house high-resolution tissue probability maps (grey matter (GM), white matter (WM) and CSF) (5) were used for the segmentation of the coregistered and brain-extracted original images. Diffeomorphic anatomical through exponentiated algebra (DARTEL) procedure (6) was applied to generate an average-shaped template within each CNV group, animal-specific flow field maps representing the transformation of the individual animal map to the average-shaped template, and warped segments reflecting GM density. For VBM, modulation of the warped segments was performed to preserve the local GM volume after normalization, while for DBM flow field maps were transformed into Jacobian determinants maps. To assess for differences in total intracranial volume, mean values of the Jacobian determinants were calculated and compared within each CNV groups using two-sample t-tests. To explore GM differences, modulated GM images were smoothed with an 0.4 mm Gaussian kernel and entered into a statistical model, once without and once with the mean value of the Jacobian determinants as covariate (SPM12). Pairwise two-sample t-tests were calculated to assess for differences between each CNV group and its respective control group. The resulting t-maps were spatially corrected for multiple comparisons using threshold-free cluster enhancement (TFCE) with a family-wise error rate (FWE) of p<0.05 (7).

**Graph analysis**

In comparison to network-based statistics, which is used to identify a cluster of connections comprising connectome associated with a group difference, graph theoretical analysis – although based on a comparable connectivity matrix – focuses on characteristics of network topology.

More specifically, brain networks are characterized by functional segregation and functional integration, with the former being the ability for specialized processing within densely interconnected groups of brain regions, and the latter being the ability to rapidly combine specialized information from distributed brain regions (8). A simple measure of segregation is clustering coefficient, which is equivalent to the fraction of the node's neighbors that are also neighbors of each other, i.e. the fraction of triangles around an individual node (8). The most common measure for integration is characteristic path length, which is calculated as the average shortest path length between all pairs of nodes in the network (8). A well-designed network can combine functionally specialized (segregation) modules with a sufficient number of intermodular links (integration), and is commonly termed as small-world network, with the respective metric being calculated as the ratio between clustering coefficient and characteristic path length (8).

Modular partitions of the individual networks were detected using the Newman algorithm (9). For all density thresholds and animals, the partitioning procedure was run 100 times and the Q-values (ratio between intra-module and extra-module connections) of all runs were averaged. The resulting Q-value represents the modularity for one animal at the given density threshold.

Degree and local clustering coefficient were assessed to explore regional alterations in network structure. Degree was calculated as the sum of edges connected to a given node. Nodes with a large degree have a high total connectivity (8). Local clustering coefficient quantifies the exchange of information within the vicinity of one node. It is computed as the average inverse shortest path length within a node’s neighborhood. Formulae of all metrics can be found in (8). Importantly, local changes in graph theoretical measures including degree and clustering coefficient might arise without widespread alterations in large clusters, implying a focal disturbance in brain function.

**Comparison between deletions**

**Network-based statistics (NBS).** As permutation tests integrated in NBS only allow a two-way ANOVA comparison with two levels per factor, respectively, we performed three two-way ANOVAs (e.g. 22q11.2 and 15q13.3 as one factor and WT vs. deletion as another factor) to compare three deletions to each other and corrected for multiple comparison. No significant NBS differences between deletions could be found.

**Seed-based analyses.** Mean time courses were extracted from each mouse, from each of the six seed regions, and were used for seed-based functional connectivity analysis (SPM12). A mean time course of the seed region was extracted from each normalized time series; then the data were smoothed by 0.6 mm (approximately 2 voxels in-plane). Correlation coefficients *r* were calculated for the extracted time courses voxel-wise and transformed to Fisher z-scores. Next, these z-score maps were fed into 2nd level analysis, using threshold-free cluster enhancement (TFCE) with a family-wise error rate (FWE) of p_FWE_ <0.05 (exemplarily contrast [1 -1 -1 1 0 0] for 22q11.2 vs. 1q21.1) to assess whole-brain functional connectivity differences between the CNV groups for a given seed (7).


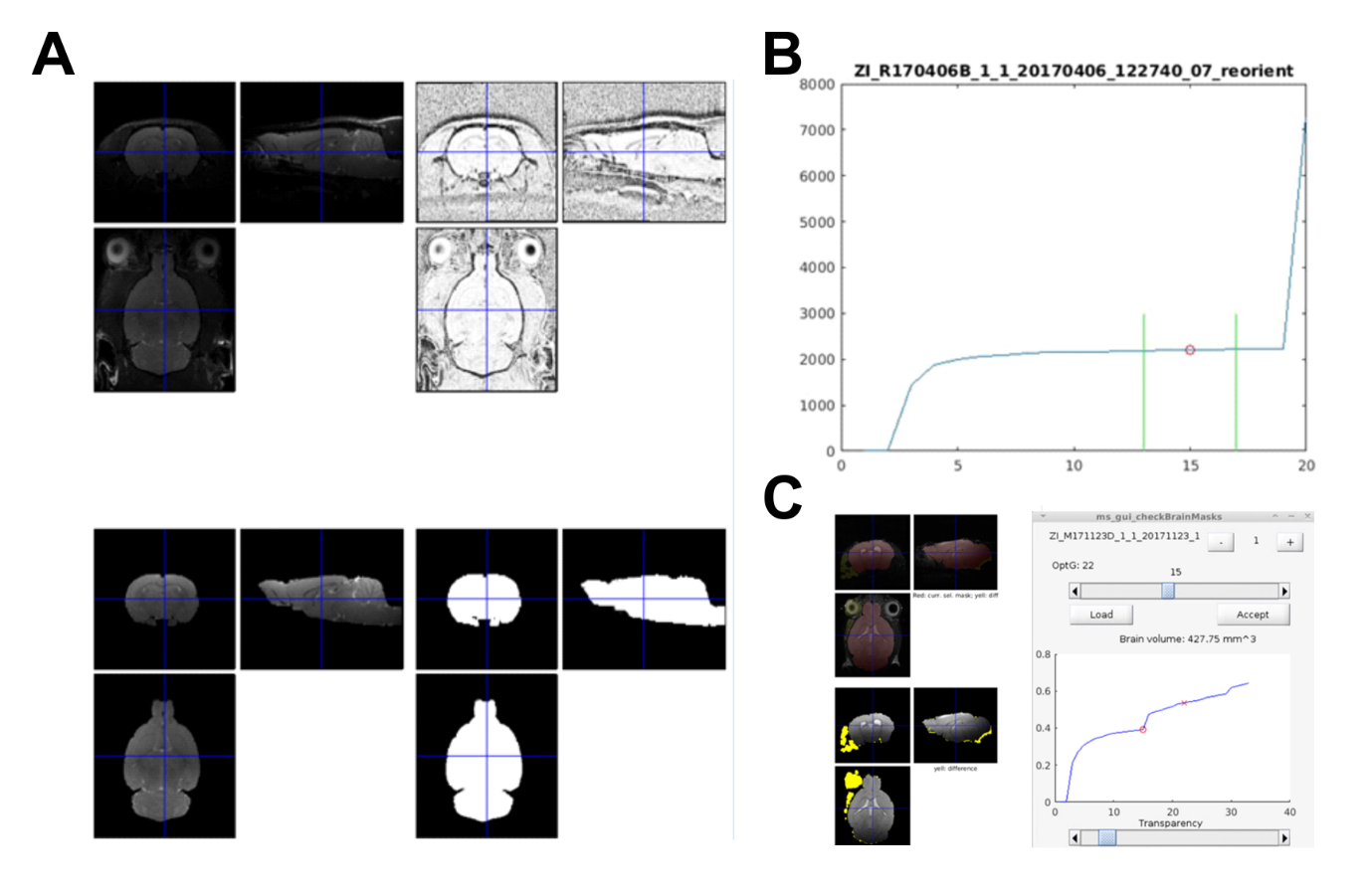


Figure S1: **Illustration of in-house brain extraction toolbox.** Starting from left top, **(A)** demonstrates the original image, the preprocessed image, the brain extraction and the mask of an exemplary animal. **(B)** shows the evolution of the volume on every step reaching a plateau. **(C)** illustrates the stepwise manual control of an exemplary brain. In this case, an additional bias correction (not shown here) improved the brain mask at the bottom.


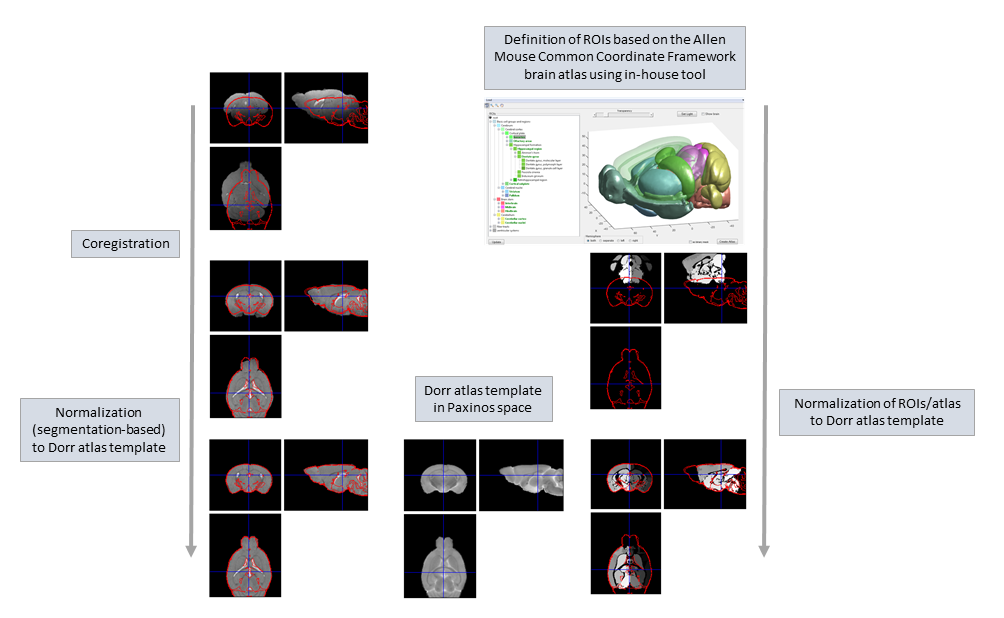


Figure S2: **Workflow illustrating the selection and normalization of regions of interests (ROI) and of our structural MRI images.** Following brain extraction and shifting, coregistration and normalization of our structural MRI images to the Dorr atlas template in the Paxinos space are illustrated on the left using exemplary data of one animal. On the right, definition of ROIs and atlas creation using an in-house tool including normalization to the Dorr atlas template in Paxinos space are illustrated. Red contours of the Dorr atlas are overlaid on all the other images demonstrating the stepwise alignment approach to the template.

**Physiological parameters**

Investigation of mean breathing and heart rates of all subgroups are illustrated in **Figure S3A**. The overall mean values of respiration (131.5.±17.5/min) and heart rate (170.9±24.4/min) were in the expected range known from the literature (10). In comparison to their respective WT littermates, most of the CNV groups demonstrated no significant differences in mean respiration and mean heart rate (**Figure S3A**, p<0.05, multiple comparison corrected), with only significant differences between 15q13.3 deletion and its WT for mean heart rate. Further, additional ANOVA analyses did not detect any differences of the physiological parameters between the CNV groups (p<0.05, multiple comparison corrected).

As physiological noise might possess a crucial confounder in resting-state functional MRI analysis, we integrated Aztec in our preprocessing pipeline, a well-established cardiorespiratory correction software for functional MRI data removing confounds and fluctuations due to cardiac pulsation, the respiration cycle, heart rate, heart rate variability and respiration volume per unit time (RVT) based on RETROICOR (11). Aztec calculates maps of explained variance, correlations and optimal lags and corrects the signal depending on the regional influence of the physiological noise. The maps of variance explained by physiological parameters of one exemplary animal are presented in **Figure S3B-C** to demonstrate the region-specific correction of the physiological confounders by the software. Bandpass filtering with a range from 0.01 to 0.1 Hz should further control for physiological noise as respiratory and cardiac frequencies are typically found within a different range – in our case between 130 and 200 Hz.

**
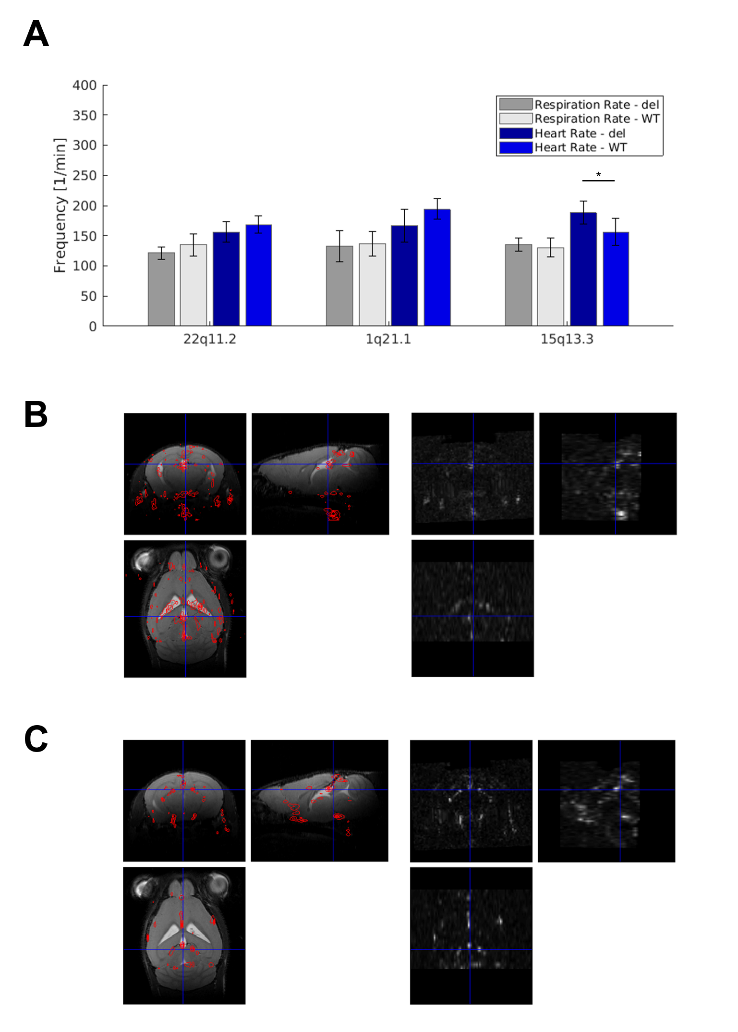
**

Figure S3: **Physiological parameters and exemplary illustration of Aztec correction. Bars (mean) with error bars (standard deviation) illustrate respiration and heart rate under medetomidine sedation (A).** Most of the CNV groups demonstrated no significant differences in mean respiration and heart rate **(A)** (p<0.05, multiple comparison corrected) compared to their respective controls, with only significant group differences between 15q13.3 deletion and its WT for mean heart rate. ANOVA analyses did not detect differences between the CNV groups (p<0.05, multiple comparison corrected). **(B-C)** illustrate the region-specific correction (maps of explained variance) for physiological noise caused by respiration **(B)** and heart rate **(C)** of one exemplary animal using Aztec software. The typical regions predominantly affected by physiological noise include ventricles for respiration and areas in vicinity to blood vessels for heart rate. *, significant difference at p<0.05 multiple comparison corrected; WT, wild type; del, deletion.


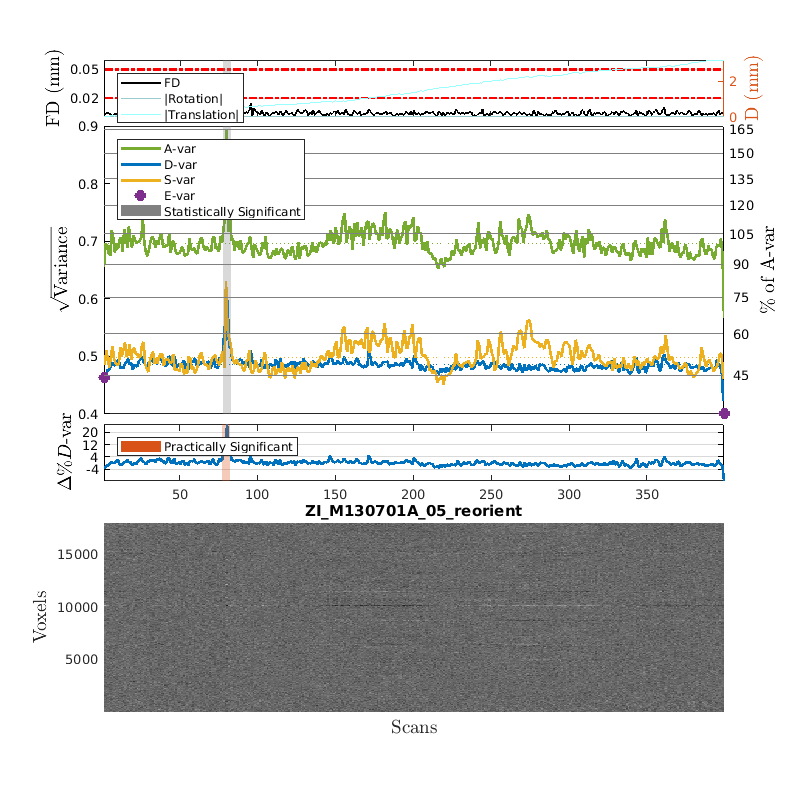


Figure S4: **DSE and DVARS inference for pre-processed data of an exemplarily (typical) animal based on** (12)**.** The upper panel shows four plots, framewise displacement (FD), the DSE plot, the percent delta-value of D-Var in percent, and an image of all brain ordinate elements. FD plots show 0.02 mm and 0.05 mm, strict and lenient thresholds, respectively. All time series plots have frames flagged as significant on the DVARS test marked gray if only statistically significant (5% Bonferroni) and orange if also practically significant (Δ%D-var>5%). Note the motion artefact around frame 80.


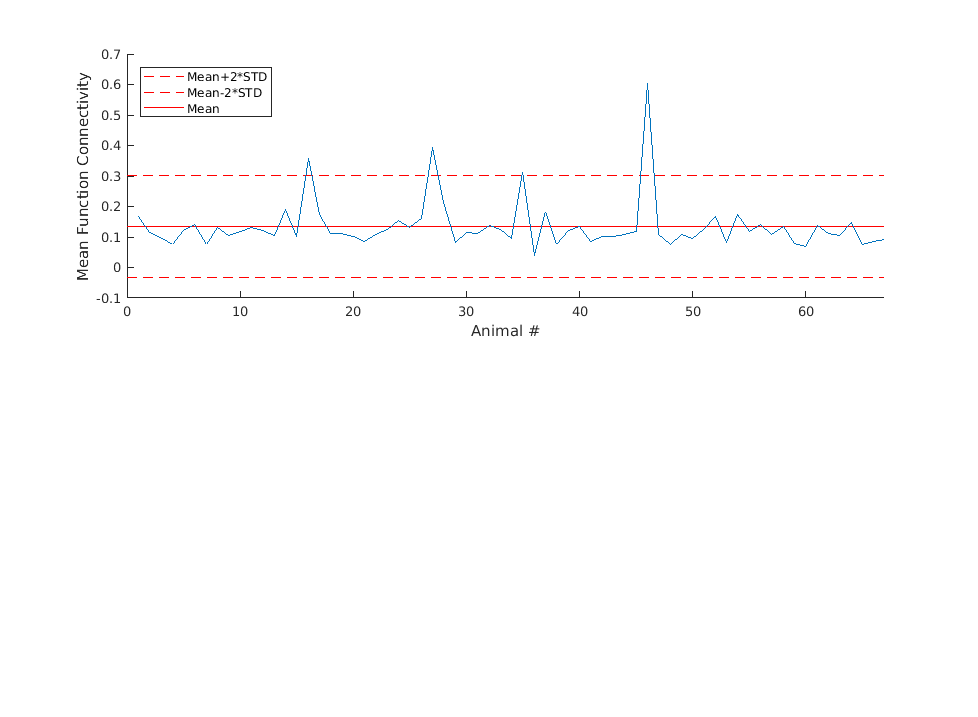


Figure S5: **Mean functional connectivity over all regions per animal.** Animals demonstrating a mean functional connectivity larger or small than twice the standard deviation from the mean of all animals were excluded from the analysis (animal #16, #27, #35 and #46).

**Results**

**Body weight**

A slightly higher body weight (6.2%, p=0.025) was observed in 15q13.3 mice (25.82 ± 0.55 g) compared to wild type mice (24.17 ± 0.39 g) at 10 weeks. In contrast, 22q11.2 mice had slightly lower weight (23.50 ± 0.36 g) compared to wild type mice (24.90 ± 0.48 g) (5.6%, p=0.032), whereas 1q21.1 had no significant difference in body weight (1q21.1 22.82 ± 0.33 g; wild type 23.18 ± 0.58 g) at 10 weeks.

**Correlation between body weights and total brain volume.**

In our morphological analysis focusing on ventricles we detected enlargement in Df(h22q11)/+ and Df(h1q21)/+ mice (trend-level), consistent with well-established morphological abnormalities in schizophrenia (13,14) and autism (15). Ventricular enlargement is also found in first-degree relatives of patients with schizophrenia (16), suggesting a genetic cause. By demonstrating this feature, our study provides a potential morphological link between the CNVs and risk for schizophrenia and autism.


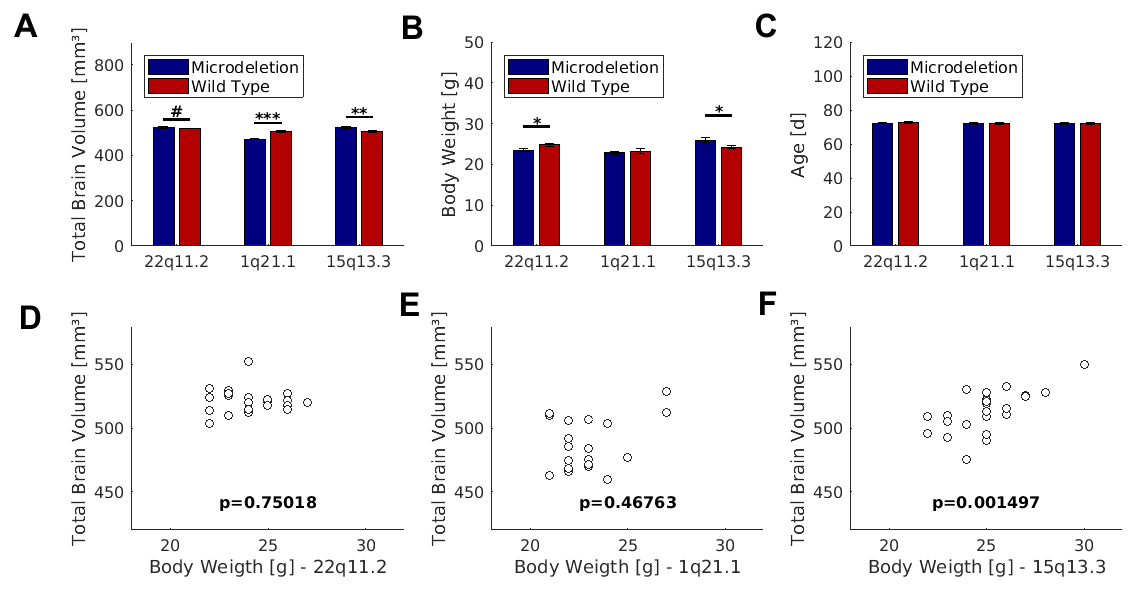


Figure S6: **Total brain volume, body weight and age in each CNV deletion and respective control group.** Error bars with standard error of the mean demonstrate differences in total brain volume **(A)**, body weight **(B)** and age **(C)**. Scatter plots **(D, E, F and G)** demonstrate significant correlation (p<0.05) between total brain volume and body weight for the 15q13.3 group **(F)**.


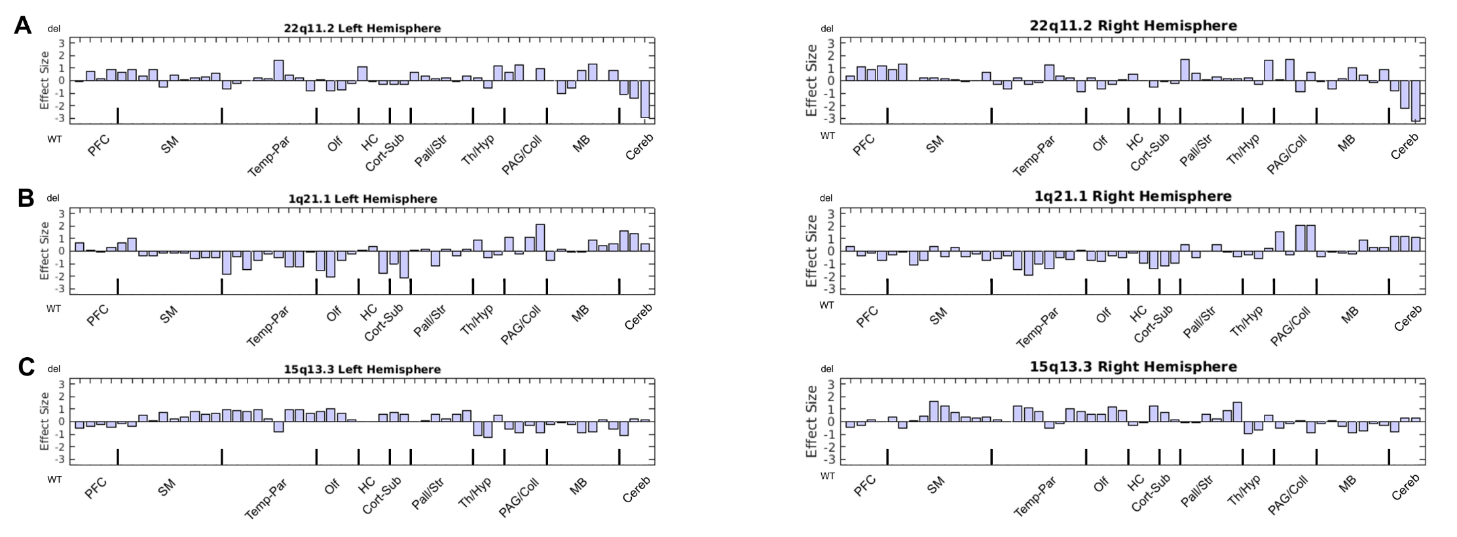


Figure S7: **Effect sizes of regional brain volume differences between Df(h22q11)/+, Df(h1q21)/+ and Df(h15q13)/+ mice and their respective controls.** Bars illustrate effects sizes (Cohen’s d) of regional **(A-C)** alterations in brain volume compared to the WT. WT, wild type; del, deletion; PFC, prefrontal cortex; SM, sensory-motor areas; Temp-Par, temporo-parietal areas; PAG/Coll, periaqueductal area and colliculi; Olf, olfactory areas; HC, hippocampal areas; Cort-Sub, cortical subplate; Pall/Str, pallidum and striatum; Th/Hyp, thalamus and hypothalamus; MB, midbrain; Cereb, cerebellum. For the abbreviations of individual brain regions see legend for Figure 2 in the manuscript.

**ROI volumetry of ventricles**

Assessing ventricular changes, we found an enlargement of the fourth ventricle in 1q21.1 deletion (p<0.05, corrected for total brain volume, **Figure S8A**) and a trend for a larger third ventricle in 22q11.2 deletion (p<0.065, corrected for total brain volume, **Figure S8C)**. Ventricular enlargement is consistent with well-established morphological abnormalities in schizophrenia (13,14) and autism (15). It is also found in first-degree relatives of patients with schizophrenia (16), suggesting a genetic cause. By demonstrating this feature, our study provides a potential morphological link between the CNVs and risk for schizophrenia and autism.


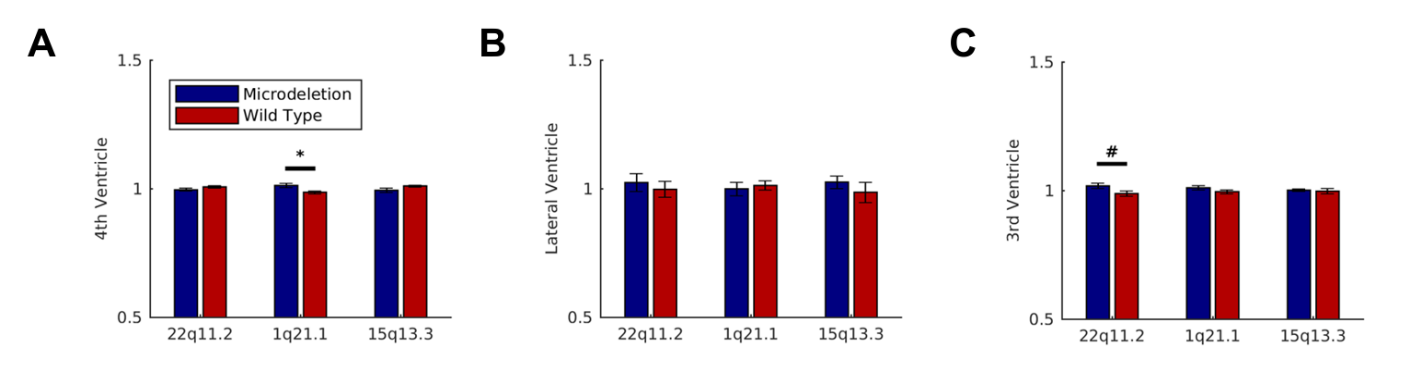


Figure S8: **Ventricular volume differences.** Bars (mean Jacobian values per ventricle) with error bars (standard error of the mean) illustrate differences in the ventricle size. *, significant at p<0.05; #, significant at trend level p<0.1.


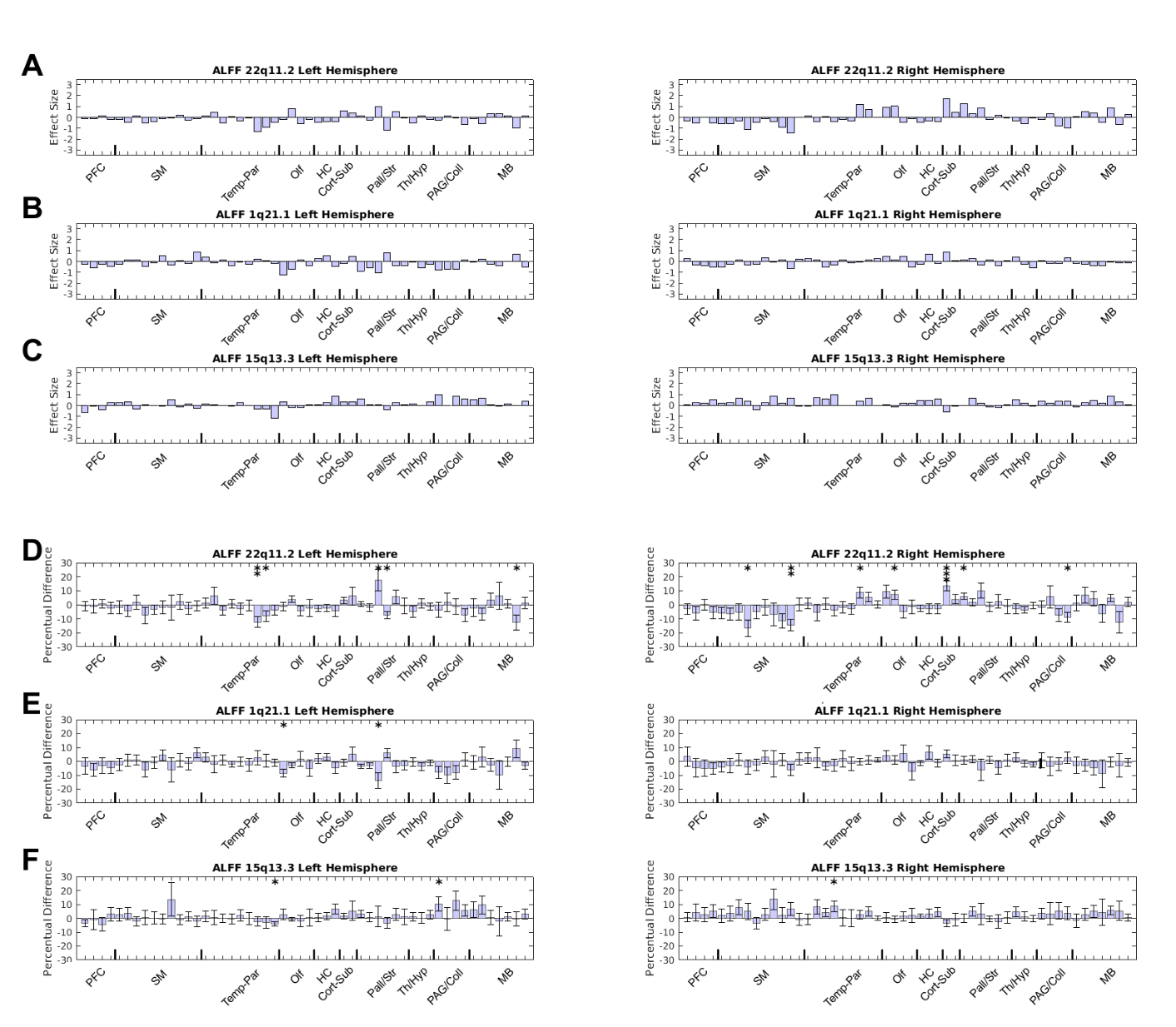


Figure S9: **Effect sizes and percentage difference of the amplitude of low frequency fluctuations (ALFF) between Df(h22q11)/+, Df(h1q21)/+ and Df(h15q13)/+ mice and their respective controls.** Bars illustrate effects sizes (Cohen’s d) in A-C of regional differences in ALFF, while bars (mean) with error bars (standard error of the mean) in D-F demonstrate percentage difference of ALFF in comparison to the respective WT. While only marginal differences were found in 15q13.3 and 1q21.1 deletion mice, 22q11.2 demonstrated most regional differences, especially in cortical and subcortical brain areas with none of them surviving multiple comparison correction. ANOVA could not detect significant similarities and differences between the CNV groups. *, significant at p<0.05; **, significant at p<0.01; ***, significant at p<0.001; §, significant at p<0.05 Bonferroni-corrected for 104 brain regions; WT, wild type; del, deletion; PFC, prefrontal cortex; SM, sensory-motor areas; Temp-Par, temporo-parietal areas; PAG/Coll, periaqueductal area and colliculi; Olf, olfactory areas; HC, hippocampal areas; Cort-Sub, cortical subplate; Pall/Str, pallidum and striatum; Th/Hyp, thalamus and hypothalamus; MB, midbrain; Cereb, cerebellum. For the abbreviations of individual brain regions see legend for Figure 2 in the manuscript.


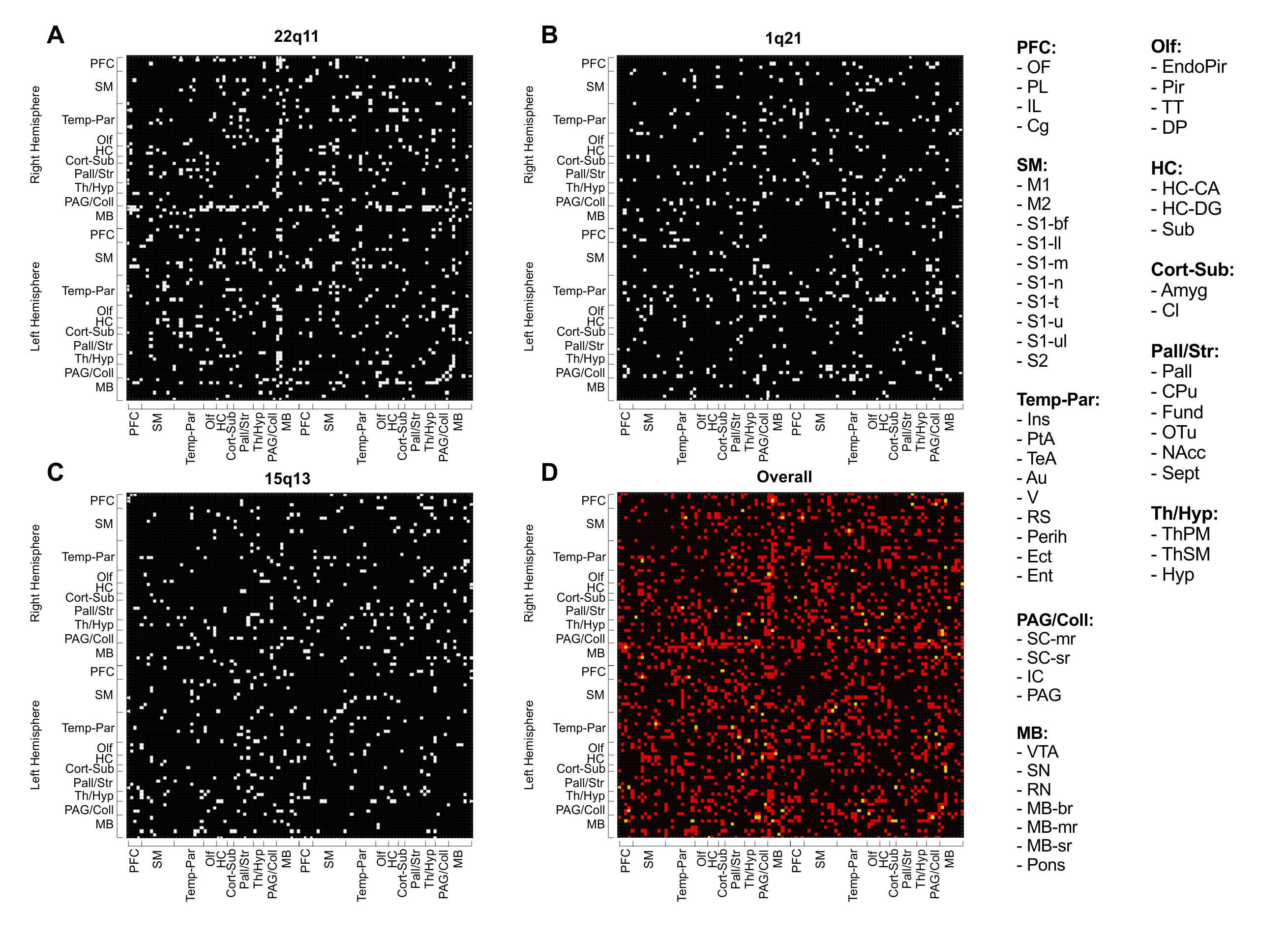


**Figure S10:** (A-C) Functional connectivity differences between each CNV deletion (22q11, 15q13 and 1q21) and its respective control group (t-test for each connection, p<0.05, uncorrected). Significant connections (p<0.05, uncorrected) are marked in white. (D) Overlap between connectivity matrices presented in plots A-C. Significant connections in one of the three plots are marked in red, in two of the three plots – yellow, and in all three plots - white. No connection was significant in all three plots.


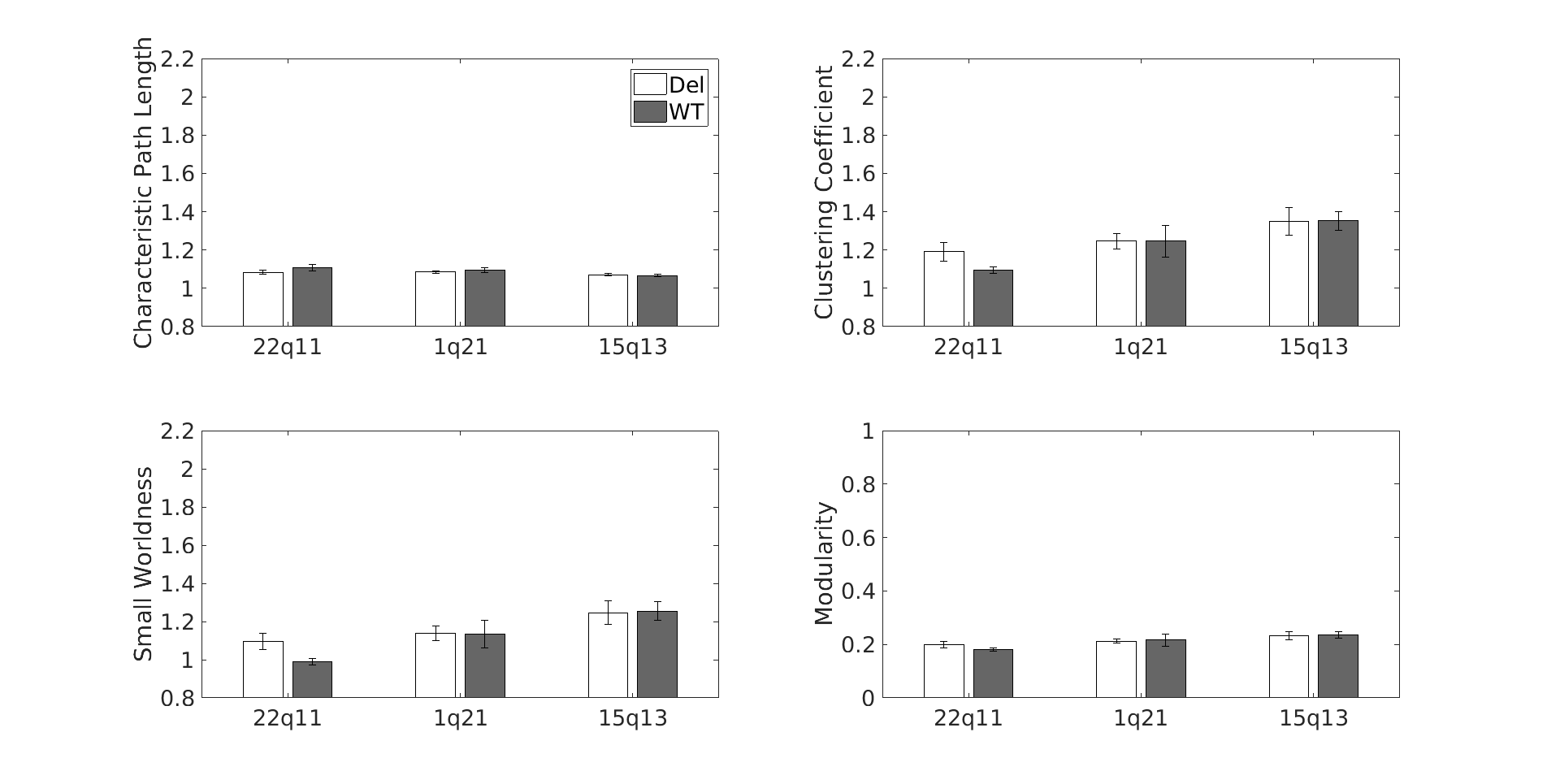


**Figure S11: Global graph metrics for 22q11.2, 15q13.3 and 1q21.1 deletion mice.**

Comparison of global graph metrics between each deletion and its respective WT group, as well as between deletions revealed no significant group differences (p>0.05).


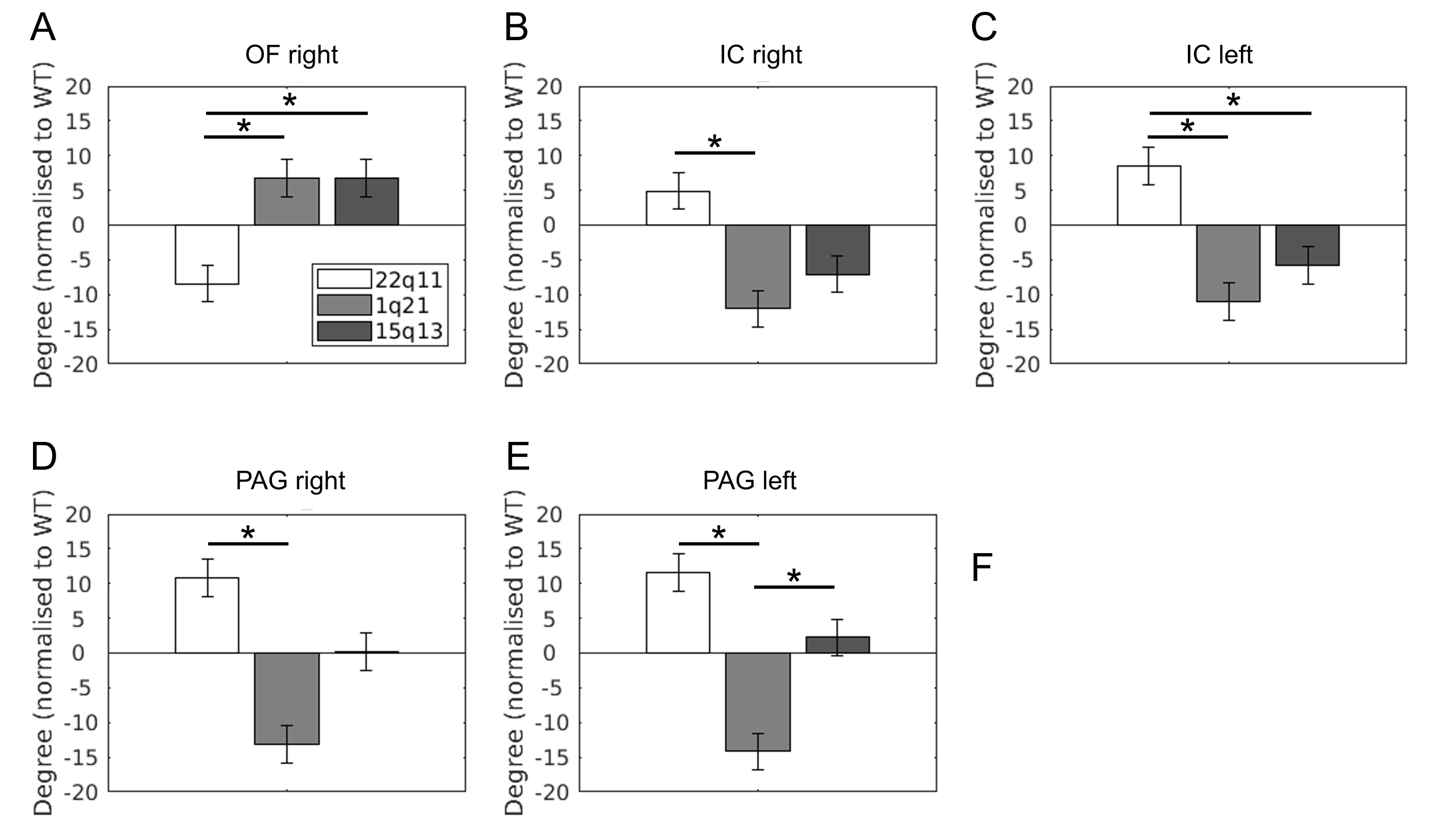


**Figure S12: Differences in degree between** **22q11.2, 1q21.1 and 15q13.3 deletion mice (normalized to WT).** Only significant results from ANOVA testing for interaction effects (group x deletion, p<0.05) of degree between the three groups are shown. Bars with error bars (standard error of the mean) depict delta values for degree **(A-F)** for 22q11.2, 1q21.1 and 15q13.3 deletion mice normalized to their respective WT. Local metrics were calculated over an area under the curve (AUC) from 16% to 50% network densities. *, significant difference at p<0.05; WT, wild type; OF, orbitofrontal cortex; IC, inferior colliculus; PAG, periaqueductal gray.

**
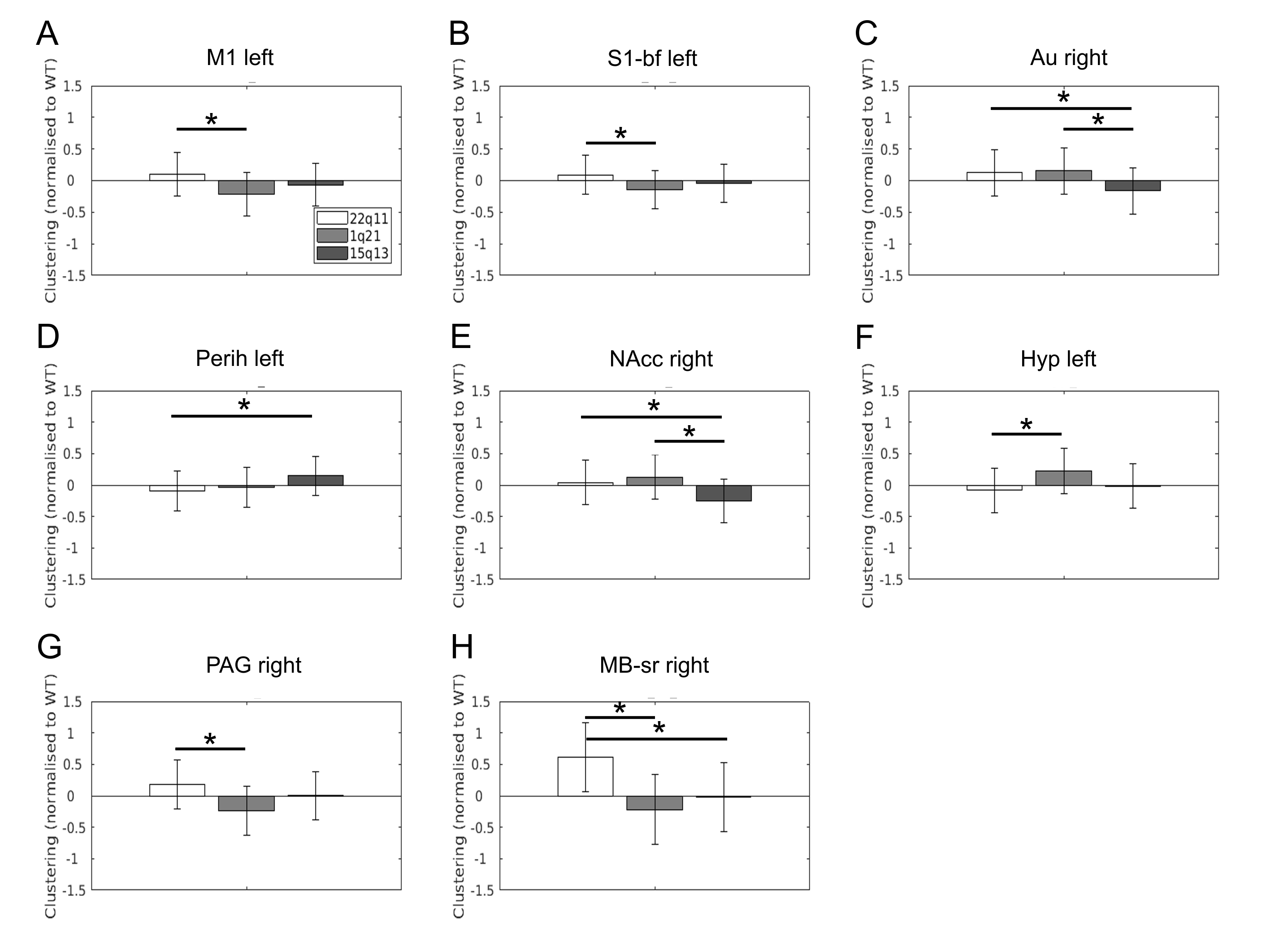
**

**Figure S13: Differences in local clustering coefficient between** **22q11.2, 1q21.1 and 15q13.3 deletion mice (normalized to WT).** Only significant results from ANOVA testing for interaction effects (group x deletion, p<0.05) of local clustering between the three groups are shown. Bars with error bars (standard error of the mean) depict delta values for local clustering **(A-F)** for 22q11.2, 1q21.1 and 15q13.3 deletion mice normalized to their respective WT. Local metrics were calculated over an area under the curve (AUC) from 16% to 50% network densities. *, significant difference at p<0.05; WT, wild type; M1, primary motor cortex; S1, primary somatosensory cortex; Aud, auditory cortex; Perih, perirhinal cortex; NAcc, nucleus accumbens; Hyp, hypothalamus; PAG, periaqueductal gray; MB, midbrain.


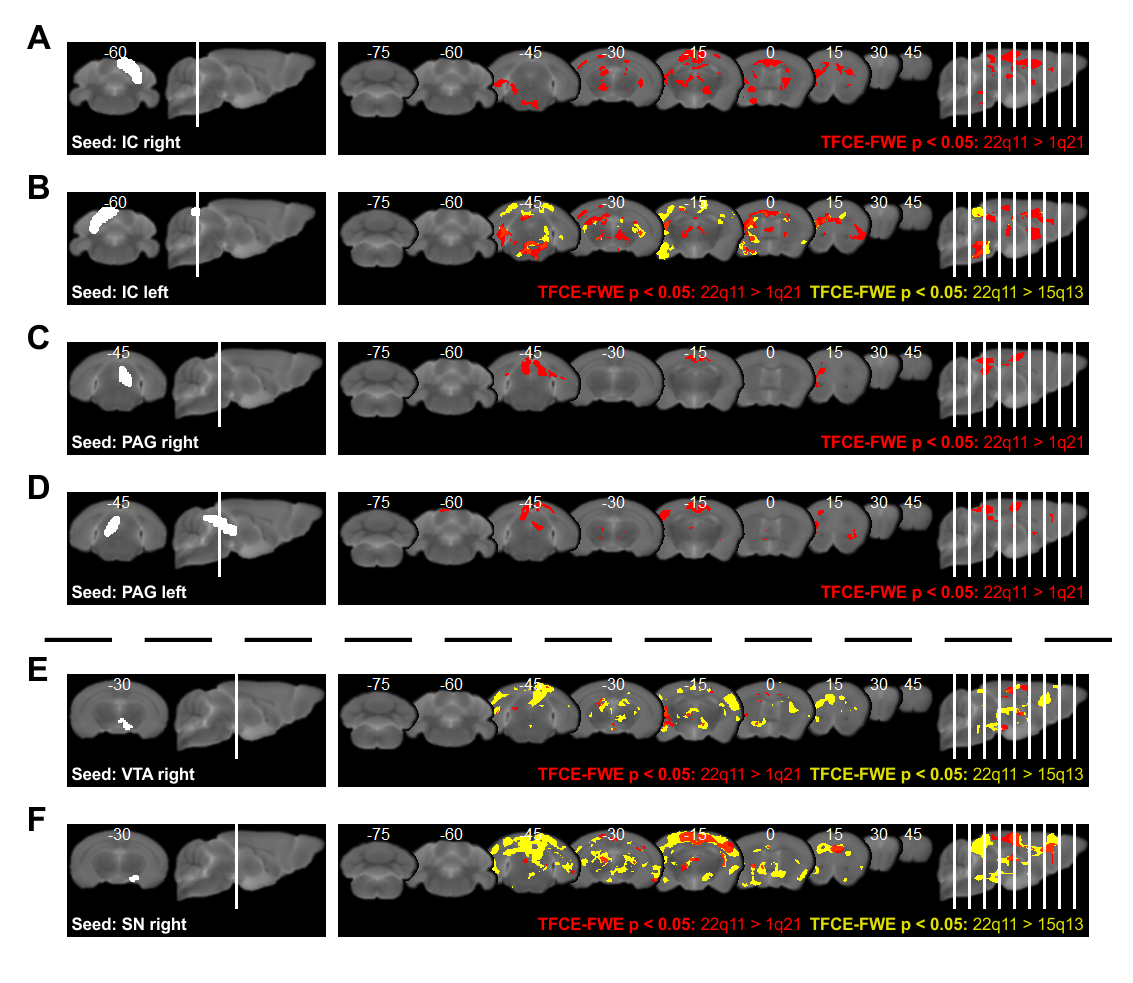


**Figure S14: Comparisons of seed-based rs-FC between** **22q11.2, 1q21.1 and 15q13.3 deletion mice.** The contrasts included the respective WT groups. White-colored seeds are located in the **(A, B)** right and left inferior colliculus (IC), **(C, D)** right and left periaqueductal gray (PAG), **(E)** right ventral tegmental area (VTA), and **(F)** right substantia nigra (SN), as illustrated on the left. Two-sample t-tests with multiple comparisons correction (threshold-free cluster enhancement with family wise error rate (TFCE-FWE) of p <0.05) show significantly higher rs-FC between IC **(A, B)**, PAG **(C,D)**, VTA **(E)**, and SN **(F)**, and multiple cortical and frontal brain regions in 22q11.2 mice compared to 1q21.1 **(red)** and 15q13.3 **(yellow)**. Coordinates are in mm to Bregma.

**REFERENCES**

(1) Chou N, Wu J, Bai Bingren J, Qiu A, Chuang KH. Robust automatic rodent brain extraction using 3-D pulse-coupled neural networks (PCNN). *IEEE Trans Image Process* 2011; 20: 2554-2564.

(2) Feo R, Giove F. Towards an efficient segmentation of small rodents brain: A short critical review. *J Neurosci Methods* 2019; 323: 82-89.

(3) Johnson JL, Padgett ML. PCNN models and applications. *IEEE Trans Neural Netw* 1999; 10: 480-498.

(4) Dorr AE, Lerch JP, Spring S, Kabani N, Henkelman RM. High resolution three-dimensional brain atlas using an average magnetic resonance image of 40 adult C57Bl/6J mice. *Neuroimage* 2008; 42: 60-69.

(5) Biedermann S et al. In vivo voxel based morphometry: detection of increased hippocampal volume and decreased glutamate levels in exercising mice. *Neuroimage* 2012; 61: 1206-1212.

(6) Ashburner J. A fast diffeomorphic image registration algorithm. *Neuroimage* 2007; 38: 95-113.

(7) Smith SM, Nichols TE. Threshold-free cluster enhancement: addressing problems of smoothing, threshold dependence and localisation in cluster inference. *Neuroimage* 2009; 44: 83-98.

(8) Rubinov M, Sporns O. Complex network measures of brain connectivity: uses and interpretations. *Neuroimage* 2010; 52: 1059-1069.

(9) Newman ME. Finding community structure in networks using the eigenvectors of matrices. *Phys Rev E Stat Nonlin Soft Matter Phys* 2006; 74: 036104.

(10) Adamczak JM, Farr TD, Seehafer JU, Kalthoff D, Hoehn M. High field BOLD response to forepaw stimulation in the mouse. *Neuroimage* 2010; 51: 704-712.

(11) van Buuren M et al. Cardiorespiratory effects on default-mode network activity as measured with fMRI. *Hum Brain Mapp* 2009; 30: 3031-3042.

(12) Afyouni S, Nichols TE. Insight and inference for DVARS. *Neuroimage* 2018; 172: 291-312.

(13) Vita A, De Peri L, Silenzi C, Dieci M. Brain morphology in first-episode schizophrenia: a meta-analysis of quantitative magnetic resonance imaging studies. *Schizophr Res* 2006; 82: 75-88.

(14) Haijma SV et al. Brain volumes in schizophrenia: a meta-analysis in over 18 000 subjects. *Schizophr Bull* 2013; 39: 1129-1138.

(15) Turner AH, Greenspan KS, van Erp TGM. Pallidum and lateral ventricle volume enlargement in autism spectrum disorder. *Psychiatry Res Neuroimaging* 2016; 252: 40-45.

(16) Lawrie SM et al. Brain structure, genetic liability, and psychotic symptoms in subjects at high risk of developing schizophrenia. *Biol Psychiatry* 2001; 49: 811-823.
